# Identification of Chemical Inhibitors of β-Catenin-Driven Liver Tumorigenesis in Zebrafish

**DOI:** 10.1371/journal.pgen.1005305

**Published:** 2015-07-02

**Authors:** Kimberley J. Evason, Macrina T. Francisco, Vladislava Juric, Sanjeev Balakrishnan, Maria del Pilar Lopez Pazmino, John D. Gordan, Sanjay Kakar, Jan Spitsbergen, Andrei Goga, Didier Y. R. Stainier

**Affiliations:** 1 Department of Pathology, University of California, San Francisco, San Francisco, California, United States of America; 2 Department of Biochemistry and Biophysics, Programs in Developmental and Stem Cell Biology, Genetics and Human Genetics, Diabetes Center, Institute for Regeneration Medicine and the Liver Center, University of California, San Francisco, San Francisco, California, United States of America; 3 Department of Cell & Tissue Biology, University of California, San Francisco, San Francisco, California, United States of America; 4 The George Williams Hooper Research Foundation, University of California, San Francisco, San Francisco, California, United States of America; 5 Department of Medicine, University of California, San Francisco, San Francisco, California, United States of America; 6 Department of Microbiology, Oregon State University, Corvallis, Oregon, United States of America; University of Pennsylvania School of Medicine, UNITED STATES

## Abstract

Hepatocellular carcinoma (HCC) is one of the most lethal human cancers. The search for targeted treatments has been hampered by the lack of relevant animal models for the genetically diverse subsets of HCC, including the 20-40% of HCCs that are defined by activating mutations in the gene encoding β-catenin. To address this chemotherapeutic challenge, we created and characterized transgenic zebrafish expressing hepatocyte-specific activated β-catenin. By 2 months post fertilization (mpf), 33% of transgenic zebrafish developed HCC in their livers, and 78% and 80% of transgenic zebrafish showed HCC at 6 and 12 mpf, respectively. As expected for a malignant process, transgenic zebrafish showed significantly decreased mean adult survival compared to non-transgenic control siblings. Using this novel transgenic model, we screened for druggable pathways that mediate β-catenin-induced liver growth and identified two c-Jun N-terminal kinase (JNK) inhibitors and two antidepressants (one tricyclic antidepressant, amitriptyline, and one selective serotonin reuptake inhibitor) that suppressed this phenotype. We further found that activated β-catenin was associated with JNK pathway hyperactivation in zebrafish and in human HCC. In zebrafish larvae, JNK inhibition decreased liver size specifically in the presence of activated β-catenin. The β-catenin-specific growth-inhibitory effect of targeting JNK was conserved in human liver cancer cells. Our other class of hits, antidepressants, has been used in patient treatment for decades, raising the exciting possibility that these drugs could potentially be repurposed for cancer treatment. In support of this proposal, we found that amitriptyline decreased tumor burden in a mouse HCC model. Our studies implicate JNK inhibitors and antidepressants as potential therapeutics for β-catenin-induced liver tumors.

## Author Summary

Liver cancer is a leading cause of cancer-related death. Genetic analysis of liver cancer has enabled classification of these tumors into subsets with unique genetic, clinical, and prognostic features. The search for targeted liver cancer treatments has been hampered by the lack of relevant animal models for these genetically diverse subsets, including liver cancers that are defined by activating mutations in the gene encoding β-catenin, an integral component of the Wnt signaling pathway. Here we describe the generation and characterization of genetically modified zebrafish expressing hepatocyte-specific activated β-catenin. We used this new zebrafish model to screen for drugs that suppress β-catenin-induced liver growth, and identified two classes of hits, c-Jun N-terminal kinase (JNK) inhibitors and antidepressants, that suppressed this phenotype. Our findings provide insights into the mechanisms by which β-catenin promotes liver tumor formation and implicate JNK inhibitors and antidepressants as potential treatments for a subset of human liver cancers.

## Introduction

Hepatocellular carcinoma (HCC) is the third leading cause of cancer-related death worldwide[1], in large part because of the paucity of effective systemic therapies. To date only one drug, the multikinase inhibitor sorafenib, has been shown to improve survival in patients with advanced HCC[2]. Genome-wide analyses of HCC have enabled the classification of these tumors into subsets and raise the possibility that the distinct signaling pathway alterations in given subgroups might be associated with different responsiveness to targeted molecular therapies[3]. A major subset of HCC is defined by activating mutations in the *CTNNB1* gene encoding β-catenin[4,5], an integral component of the canonical Wnt signaling pathway[6]. Studies of human liver tumors suggest that β-catenin mutation may be an early or initiating event in some HCC[4,7,8].

Vertebrate models that recapitulate the specific signaling pathway perturbations that occur in HCC, such as the Wnt/β-catenin signaling pathway, could help further define mechanisms by which these pathways promote HCC. Such models could be highly useful in the identification and characterization of targeted systemic therapies unique to each tumor type. Loss-of-function mutations in *APC*, which result in increased β-catenin activity, increase hepatic neoplasia in both mice[9] and zebrafish[10]. Interestingly, although expression of activated β-catenin can induce some types of mouse tumors, including intestinal and prostatic neoplasms[6], and mutant β-catenin enhances murine liver tumorigenesis in response to carcinogen treatment[11] and H-Ras co-expression[12], activated β-catenin is not sufficient to induce HCC in mice[6,12,13]. Thus, the use of mouse models to study events in liver tumor formation that may be uniquely and specifically related to β-catenin mutation is limited. The zebrafish represents an excellent model system for studies of human cancer, given its powerful genetics and amenability to chemical screens[14] and the histologic and genetic similarities between zebrafish and human tumors[15]. At early developmental stages, zebrafish larvae are translucent, and internal organs such as the liver and heart can be readily observed in live animals using a dissecting microscope.

To investigate mechanisms by which activated β-catenin signaling promotes liver tumor formation and to identify potential therapeutics for these cancers, we generated transgenic zebrafish expressing hepatocyte-specific activated β-catenin (*Tg(fabp10a*:*pt-β-cat)* zebrafish). As adults, these animals show increased liver size, decreased survival, and histologic abnormalities similar to human HCC. The presence of a larval phenotype enabled us to use these zebrafish in a whole-organism chemical screen, in which we tested 960 drugs and identified eight compounds, including two c-Jun N-terminal kinase (JNK) inhibitors and two antidepressants (a tricyclic antidepressant (TCA) and a selective serotonin reuptake inhibitor (SSRI)), that suppressed β-catenin-induced liver enlargement. The β-catenin-specific growth-inhibitory effect of targeting JNK was conserved in human liver cancer cells. Furthermore, activated β-catenin was associated with JNK pathway hyperactivation in zebrafish and in human HCC. Focusing additional chemical screening on serotonergic ligands, we identified two other antidepressants (a TCA and a tetracyclic antidepressant) that suppressed β-catenin-induced liver enlargement. All of our antidepressant hits are FDA-approved, raising the exciting possibility that these drugs or related compounds could potentially be repurposed for cancer treatment. Supporting the hypothesis that these medications might have anti-tumor effects in mammals, we found that the TCA amitriptyline decreased tumor burden in a mouse HCC model. Together, our results show that *Tg(fabp10a*:*pt-β-cat)* zebrafish represent a useful tool for studies of β-catenin-driven liver tumorigenesis, enabling insights into mechanism and potential therapeutics for liver cancers with β-catenin mutations, including β-catenin-activated human HCC.

## Results

### Zebrafish expressing activated β-catenin develop HCC

As a first step to investigate mechanisms by which activated β-catenin promotes liver tumorigenesis, we generated zebrafish that express a mutated form of *Xenopus laevis ctnnb1/β-catenin* (*pt-β-cat*)[16] under the control of hepatocyte-specific regulatory sequences from the zebrafish gene *fabp10a*[17](S1A Fig). *Xenopus* β-catenin is highly similar to human and zebrafish β-catenin. Its *in vivo* activity has been extensively studied[16], and its transcript can be distinguished from endogenous zebrafish transcripts. *pt-β-cat* contains four point mutations affecting putative phosphorylation sites (S33A, S37A, T41A, and S45A); these mutations activate β-catenin by inhibiting its phosphorylation and subsequent degradation, and the affected codons are among those most commonly mutated in human HCC[18]. We engineered the transgene to also encode a fluorescent protein downstream of lens-specific regulatory sequences (*cryaa*:Venus), such that transgenic zebrafish can be identified by 3 days of age (days post fertilization, dpf) based on eye color[19,20]. We generated three independent lines of *Tg(fabp10a*:*pt-β-cat*, *cryaa*:*Venus)* zebrafish (hereafter used interchangeably and named *Tg(fabp10a*:*pt-β-cat)*), which showed similar phenotypes, supporting the hypothesis that the resulting changes are due to expression of *pt-β-cat* and not to disruption of another gene during transgenesis. Transgenic zebrafish exhibited *Xenopus ctnnb1* mRNA expression in their livers (S1B Fig), while *Xenopus ctnnb1* was undetectable in non-transgenic zebrafish (S1B Fig). Zebrafish *ctnnb1* mRNA levels were not significantly different in transgenic versus non-transgenic livers (S1C Fig), suggesting that endogenous *ctnnb1* transcription was not suppressed by the presence of the transgene. Further, *Tg(fabp10a*:*pt-β-cat)* zebrafish exhibit nuclear β-catenin localization and Wnt reporter activation[21](S1 Table) in their hepatocytes by 5–6 dpf (S1D–S1G Fig), indicating hepatocyte-specific expression of activated β-catenin.

**Fig 1:**
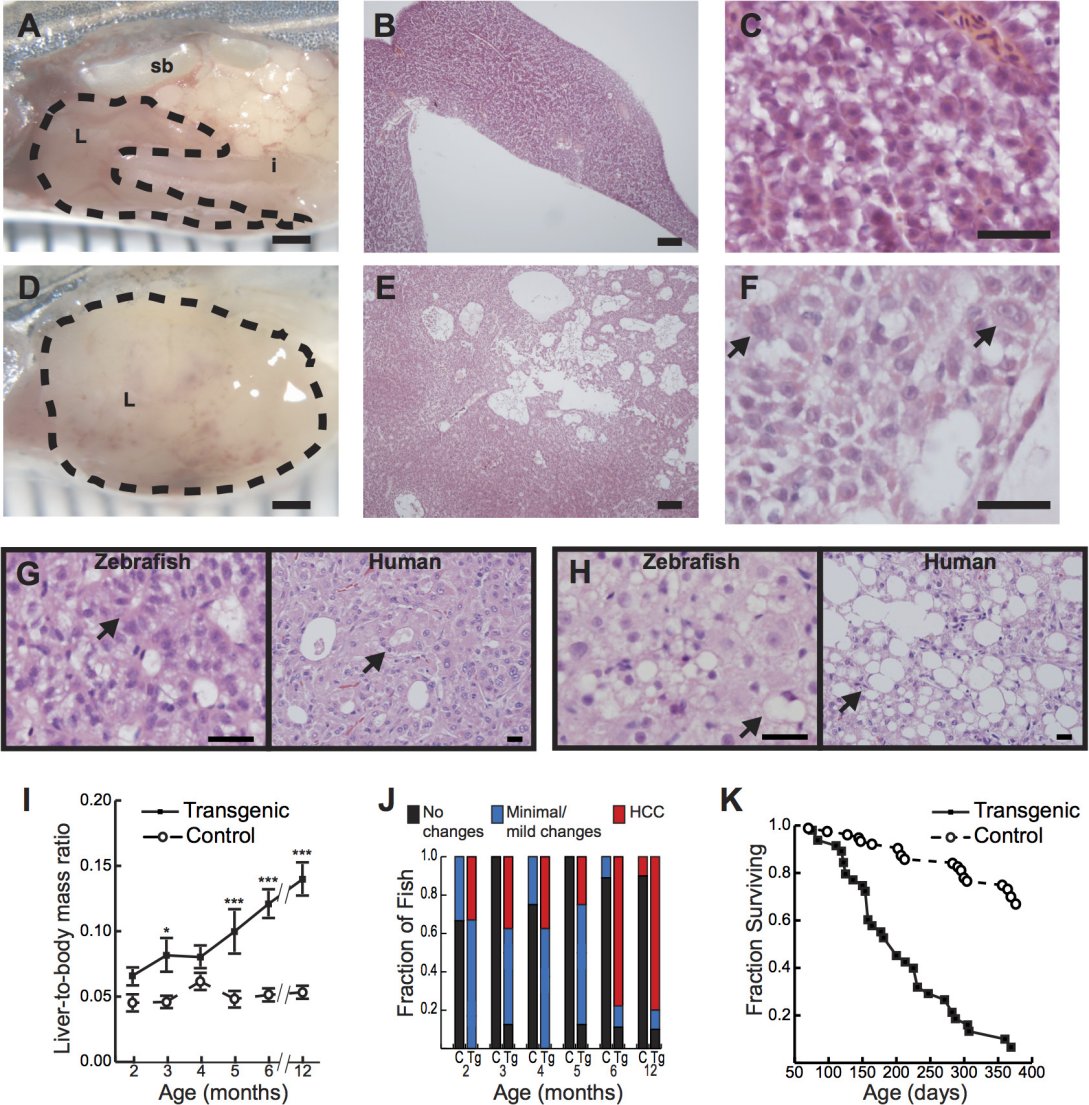
Hepatocyte-specific expression of activated β-catenin results in liver enlargement, hepatocellular carcinoma (HCC), and decreased survival in adult zebrafish. (**A-C**) Control 4-month-old zebrafish showing the liver (L, outlined) positioned in the body cavity near the intestine (i) and swim bladder (sb)(**A**). Sections show an orderly arrangement of hepatocytes (**B-C**). (**D-F**) Transgenic 4-month-old zebrafish showing an enlarged liver (**D**) with disorganized architecture (**E**) and atypical cells (arrows, **F**). (**G-H**) Transgenic 6-month-old (**G**) or 4-month-old (**H**) zebrafish and human HCC showing architectural disruption with scattered pseudoglands (arrows, **G**) and evidence of intracellular lipid accumulation (arrows, **H**). Hematoxylin and eosin stains; scale bars, 1 mm (**A, D**), 100 μm (**B, E**), 25 μm (**C, F**), and 20 μm (**G, H**). (**I**) Graph showing average liver size normalized to total body mass, ± standard error of the mean (SEM). Asterisks indicate p-values for ANOVA comparing transgenic zebrafish (N = 56) to control siblings (N = 51) at the same time point: *, p<0.05; ***, p<0.001. (**J**) Livers of transgenic zebrafish (Tg, N = 49) and control siblings (C, N = 37) were examined microscopically, and architectural and cytological changes were scored. HCC was significantly more common in transgenic zebrafish than in controls (p<0.001, Fisher’s exact test). (**K**) Kaplan-Meier survival curves comparing adult survival of transgenic zebrafish (N = 51) and control siblings (N = 85); p<0.001, logrank test.

By 4 to 5 months of age, *Tg(fabp10a*:*pt-β-cat)* zebrafish showed dramatically enlarged livers with increased vasculature and/or discoloration involving 1 or more lobes (Fig 1A and 1D). Microscopically, these transgenic zebrafish livers showed patchy involvement by neoplastic change, with areas of architectural disruption (Fig 1B, 1E, and S2A) and atypical cytological features including nuclear enlargement and nuclear contour irregularities (Fig 1C, 1F, and S2B–S2D Fig). Importantly, similar architectural and cytological abnormalities have been used to diagnose HCC in other transgenic zebrafish models[22] and represent defining features of well- to moderately-differentiated human HCC (S2 Table). For example, zebrafish livers with activated β-catenin often showed abnormal circular gland-like arrangements of hepatocytes (Fig 1G) resembling the pseudoglands seen in many human HCCs. Like human liver tumors, zebrafish samples occasionally showed evidence of intracellular lipid accumulation, with fat vacuoles displacing hepatocyte nuclei to the edge of the cell (Fig 1H). Overall, the microscopic appearance of *Tg(fabp10a*:*pt-β-cat)* zebrafish livers was diverse, mimicking the variety of architectural and cytological features that is seen in human HCC. Thus, our data indicate that *Tg(fabp10a*:*pt-β-cat)* zebrafish recapitulate morphologic features of human HCC.

To determine the incidence of these gross and microscopic alterations, we examined *Tg(fabp10a*:*pt-β-cat)* zebrafish and non-transgenic control siblings at 1 to 6 month intervals. We found that liver size relative to total body mass was significantly increased in *Tg(fabp10a*:*pt-β-cat)* zebrafish compared to controls at multiple time points (Fig 1I). *Tg(fabp10a*:*pt-β-cat)* zebrafish had lower body mass at most time points (S3 Fig and S3 Table), although this difference was only statistically significant at 4 months post fertilization (p<0.01, 2-way ANOVA). Livers were analyzed for HCC by a board-certified pathologist with subspecialty fellowship training in liver pathology (K.J.E.) using histologic criteria based on architectural and cytological abnormalities (S2 Table) as described previously[22]. By 2 months post fertilization (mpf), 33% of *Tg(fabp10a*:*pt-β-cat)* zebrafish developed HCC in their livers, and this percentage increased to 78% and 80% at 6 and 12 mpf, respectively (Fig 1J). Overall, while 25 out of 49 (51%) *Tg(fabp10a*:*pt-β-cat)* livers showed HCC, only 1 out of 37 (2.7%) control livers showed such features (Fig 1J; p<0.001, Fisher’s exact test). Furthermore, the median adult survival of *Tg(fabp10a*:*pt-β-cat)* zebrafish was decreased to 200 days compared to greater than 393 days in non-transgenic control siblings (Fig 1K; p<0.001, logrank test). Together, these results show that activated β-catenin is sufficient to promote HCC in zebrafish.

Genetic instability and aneuploidy are hallmarks of many human cancers including HCC; DNA aneuploidy, measured by flow cytometric measurement of DNA content, is associated with malignancy in human[23] and zebrafish[24]. Therefore, we next sought to examine whether *Tg(fabp10a*:*pt-β-cat)* livers showed evidence of DNA aneuploidy by flow cytometric analysis. We found that while no control animals (0/8) showed DNA aneuploidy by flow cytometry (S4A–S4D Fig), 5 out of 14 (36%) *Tg(fabp10a*:*pt-β-cat)* zebrafish showed evidence of DNA aneuploidy (S4E–S4H Fig).

To further characterize our model, we used microarray analysis to compare gene expression in *Tg(fabp10a*:*pt-β-cat)* zebrafish livers with HCC to that of non-transgenic control sibling livers (deposited in NCBI's Gene Expression Omnibus[25] and accessible through GEO Series accession number GSE68124). As expected, several known Wnt/β-catenin target genes showed increased expression in *Tg(fabp10a*:*pt-β-cat)* zebrafish livers with HCC compared to non-transgenic control sibling livers, including *myca*[26] (1.9-fold increase), *lef1*[27] (3.3-fold increase), *pparda*[28] (2.0-fold increase), and *sp5*[29] (2.1-fold increase). We performed Ingenuity Pathways Analysis (IPA) (Ingenuity Systems, www.ingenuity.com) of differentially expressed genes (S5 Fig). IPA revealed that “Cancer” (p-value 4.14E-10 to 6.56E-03) and “Gastrointestinal Disease” (p-value 3.53E-08 to 3.32E-03) were the two most significantly affected “Diseases and Disorders” in *Tg(fabp10a*:*pt-β-cat)* zebrafish livers.

**Fig 2:**
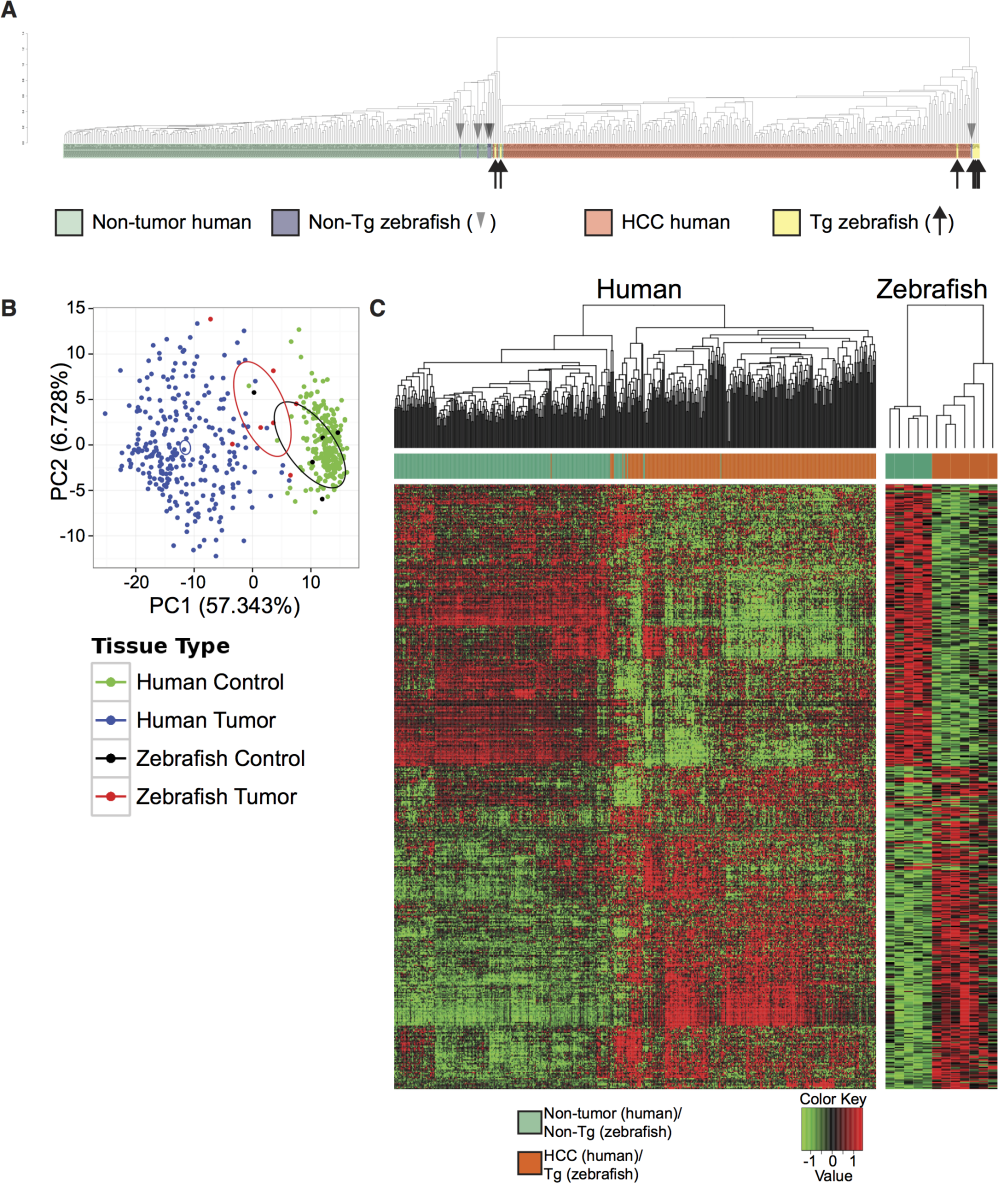
Cross-species comparison of *Tg(fabp10a*:*pt-β-cat)* zebrafish and human HCC. (**A-B**) Analyses of *Tg(fabp10a*:*pt-β-cat)* zebrafish RNA-seq data and publicly available human microarray data based on a subset of 283 orthologous gene pairs that are significantly dys-regulated in human HCC samples when compared to adjacent non-tumor human liver samples. (**A**) Dendrogram illustrating hierarchical clustering of merged human and zebrafish samples, showing that 5 of 7 transgenic zebrafish livers cluster with human HCC and 4 of 5 non-transgenic zebrafish livers cluster with non-tumor human liver. (**B**) Principal component analysis of *Tg(fabp10a*:*pt-β-cat)* zebrafish livers, non-transgenic control livers, human HCC, and non-tumor human liver. Ellipses indicate the 95% confidence intervals of the first two principal components for the respective groups. (**C**) Heatmaps of human and zebrafish orthologs that are significantly dys-regulated in the same direction in both human HCC samples and *Tg(fabp10a*:*pt-β-cat)* animals when compared to their non-tumor counterparts (FDR < 0.05).

We next sought to more deeply investigate possible similarities in gene expression between *Tg(fabp10a*:*pt-β-cat)* zebrafish livers and human liver cancer. We measured gene expression in *Tg(fabp10a*:*pt-β-cat)* zebrafish livers with HCC and non-transgenic control sibling livers by RNA-seq and performed combined clustering analysis of these zebrafish samples with 268 human HCC samples and 243 adjacent non-tumor human liver tissue (GSE25097)[30–32]; we based our clustering on 283 differentially-expressed genes in human HCC versus human non-tumor liver (absolute log fold-change (logFC) greater than or equal to 2 and false-discovery rate (FDR) less than 0.05) (S1 Dataset). We found that 5 out of 7 (71%) *Tg(fabp10a*:*pt-β-cat)* zebrafish liver samples clustered with human HCC, and 4 out of 5 (80%) non-transgenic zebrafish liver samples clustered with non-tumor human liver samples (Fig 2A). The gene expression similarities between *Tg(fabp10a*:*pt-β-cat)* zebrafish liver tumors and human HCC are corroborated by a visualization of the first two principal components (Fig 2B). Next, we identified 485 genes that were significantly dys-regulated in the same direction in both human HCC (GSE25097) and *Tg(fabp10a*:*pt-β-cat)* zebrafish livers, when compared to the corresponding non-tumor/non-transgenic controls, at an FDR less than 0.05 (Fig 2C, S1 Dataset). Pathway enrichment analyses of these genes highlighted several pathways that are relevant to cancer, including DNA replication, DNA repair, cell proliferation, telomeres, and cell motility and adhesion (S4 Table, S1 Dataset). Together, these results suggest that there are striking transcriptional similarities between *Tg(fabp10a*:*pt-β-cat)* zebrafish liver tumors and human HCC, and that these similarities may be important for liver tumorigenesis in both species.

### Chemical screen for suppressors of β-catenin-induced liver enlargement

**Fig 3:**
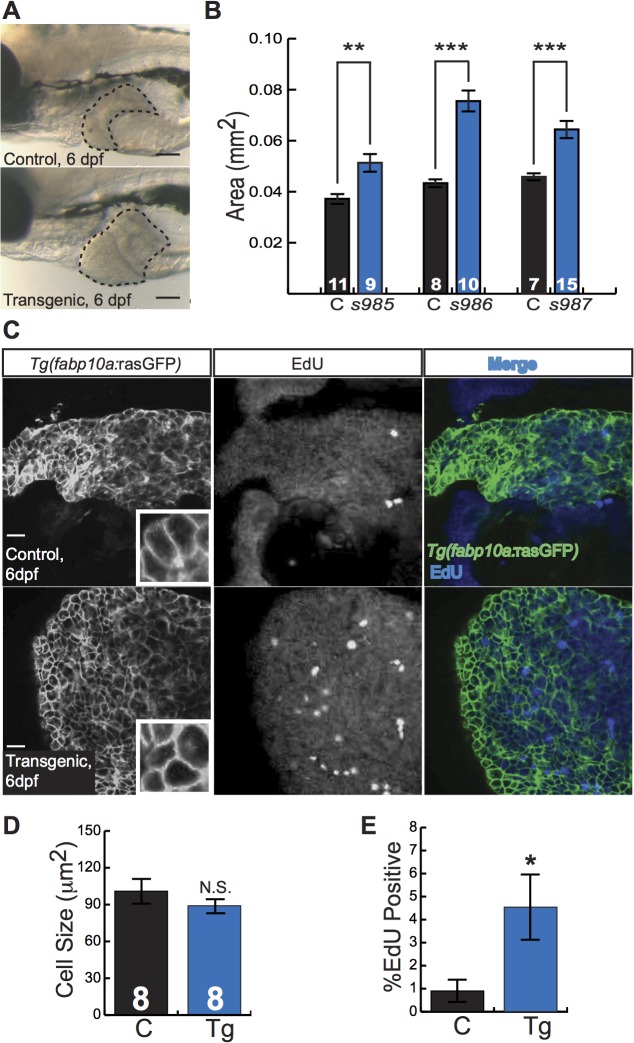
Activated β-catenin causes larval liver enlargement and increased hepatocyte proliferation. (**A**) Brightfield images of control sibling and transgenic 6-day-old fixed larvae. Livers are outlined. Scale bars, 100 μm. (**B**) Graph showing average liver size ± SEM of 6-day-old larvae from three different transgenic lines (*s985*, *s986*, *s987*) compared to control siblings (C). N values are shown above the x-axis. Asterisks indicate p-values for 2-way ANOVA comparing transgenic zebrafish to control siblings in the same experiment: **, p<0.01; ***, p<0.001. (**C**) Immunofluorescence images of 6-day-old control and transgenic larvae, highlighting hepatocyte cell membranes (*Tg(fabp10a*:rasGFP)) and proliferating cells (EdU). Scale bars, 20 μm. Inset photos are 4X magnifications. (**D-E**) Graphs showing average cell size ± SEM (**D**) and percent of hepatocytes that were EdU positive (**E**). N values, which are the same for both graphs, appear above the x-axis in **D**; samples were compared using the Student’s t-test. *, p<0.05.

Zebrafish larvae are small, translucent, and readily absorb compounds from their culture water, facilitating their use in chemical screens[14]. To investigate the utility of *Tg(fabp10a*:*pt-β-cat)* larvae as a tool for chemical screening, we examined liver development in these animals. Already at 5 to 6 dpf, *Tg(fabp10a*:*pt-β-cat)* livers from all three transgenic lines were 40–70% larger than the livers of control siblings (Fig 3A and 3B). Given the well-established role of activated β-catenin in driving liver growth and proliferation[1,6], we hypothesized that the increase in liver size seen in zebrafish expressing activated β-catenin was due to increased cell proliferation. To test this hypothesis, we performed 5-ethynyl-2´-deoxyuridine (EdU) labeling and cell size measurements. We found that *Tg(fabp10a*:*pt-β-cat)* livers showed 130% more EdU labeling than control sibling livers, while hepatocyte size was not significantly altered (Fig 3C–3E).

To identify druggable pathways that mediate β-catenin-mediated liver enlargement, we next established a protocol for small-molecule chemical screening using *Tg(fabp10a*:*pt-β-cat)* larval zebrafish (S6 Fig). For this chemical screen, we also employed *Tg(fabp10a*:*EGFP)* zebrafish[17], which express liver-specific green fluorescent protein by 3 dpf, thus facilitating rapid visual assessment of liver size in live animals. Three 3-dpf zebrafish were cultured in each well with drug or vehicle control for 3 days. At 6 dpf we scored liver size using a fluorescence dissecting microscope on a scale from 0 to 4. Concurrently, animals were examined for evidence of drug toxicity including pericardial edema or death. Drugs that substantially decreased average liver size of *Tg(fabp10a*:*pt-β-cat); Tg(fabp10a*:*EGFP)* zebrafish in 2 out of 2 wells (both drug-containing wells) without signs of toxicity were considered potential hit compounds. We then performed a secondary validation screen of these potential hit compounds, using similar parameters with more animals.

**Fig 4:**
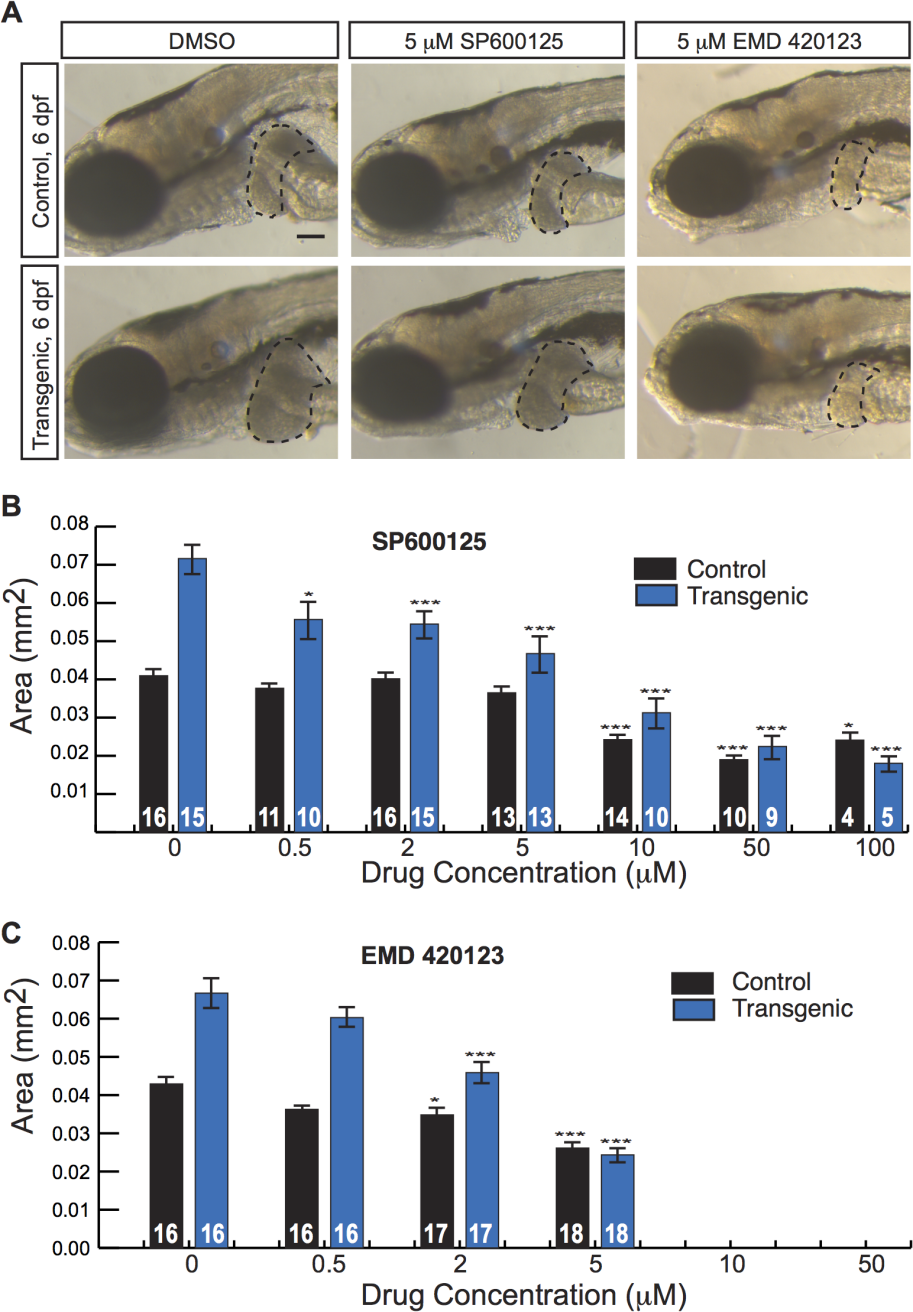
JNK inhibitors suppress larval liver enlargement caused by activated β-catenin. (**A**) Brightfield images of control sibling and transgenic 6-day-old fixed larvae, treated with DMSO or JNK inhibitors. Livers are outlined. Scale bar, 100 μm. (**B-C**) Graphs showing average liver size ± SEM of 6-day-old control sibling and transgenic larvae treated for 3 days with SP600125 (**B**) or EMD 420123 (**C**) at the indicated dosages. N values are shown above the x-axis. Asterisks indicate p-values for 2-way ANOVA comparing drug-treated zebrafish to DMSO-treated siblings with the same genotype: *, p<0.05; ***, p<0.001.

As many anti-cancer agents are protein kinase inhibitors, we next applied our screening platform to a commercially available kinase inhibitor library. We tested 160 compounds and identified five drugs that decreased liver size in *Tg(fabp10a*:*pt-β-cat)* zebrafish without causing obvious toxicity (S5 Table). We selected SP600125 and EMD 420123 for additional follow-up studies because they were the only 2 of the 5 hits among the kinase inhibitors with a shared mechanism of action. SP600125 is a well-established JNK inhibitor[33]. EMD 420123 also inhibits JNK, albeit with a substantially higher IC50 *in vitro*[33]. At doses greater than or equal to 10 μM, both drugs caused edema and/or death. All larvae (34/34 and 27/27) treated with 10 μM or 50 μM EMD 420123 died. Five out of 31 and 7/31 larvae treated with 10 μM SP600125 died or showed edema, respectively; 2/21 and 17/21 larvae treated with 50 μM SP600125 died or showed edema; and 2/10 and 8/10 larvae treated with 100 μM SP600125 died or showed edema. At lower doses, both SP600125 and EMD 420123 caused a dose-dependent decrease in liver size in *Tg(fabp10a*:*pt-β-cat)* larvae (Fig 4A–4C). Although EMD 420123 also decreased liver size in non-transgenic animals, the magnitude of this effect was less substantial (20–40% decrease at 2–5 μM) than the effect on zebrafish expressing activated β-catenin (30–60% decrease at 2–5 μM). Furthermore, at doses up to 5 μM, SP600125 did not affect liver size in non-transgenic animals, suggesting that the decrease in liver size we observed with this compound was related to specific effects in the context of activated β-catenin and not due to non-specific toxicity.

### Activated β-catenin is associated with JNK activity in larval and adult zebrafish

**Fig 5:**
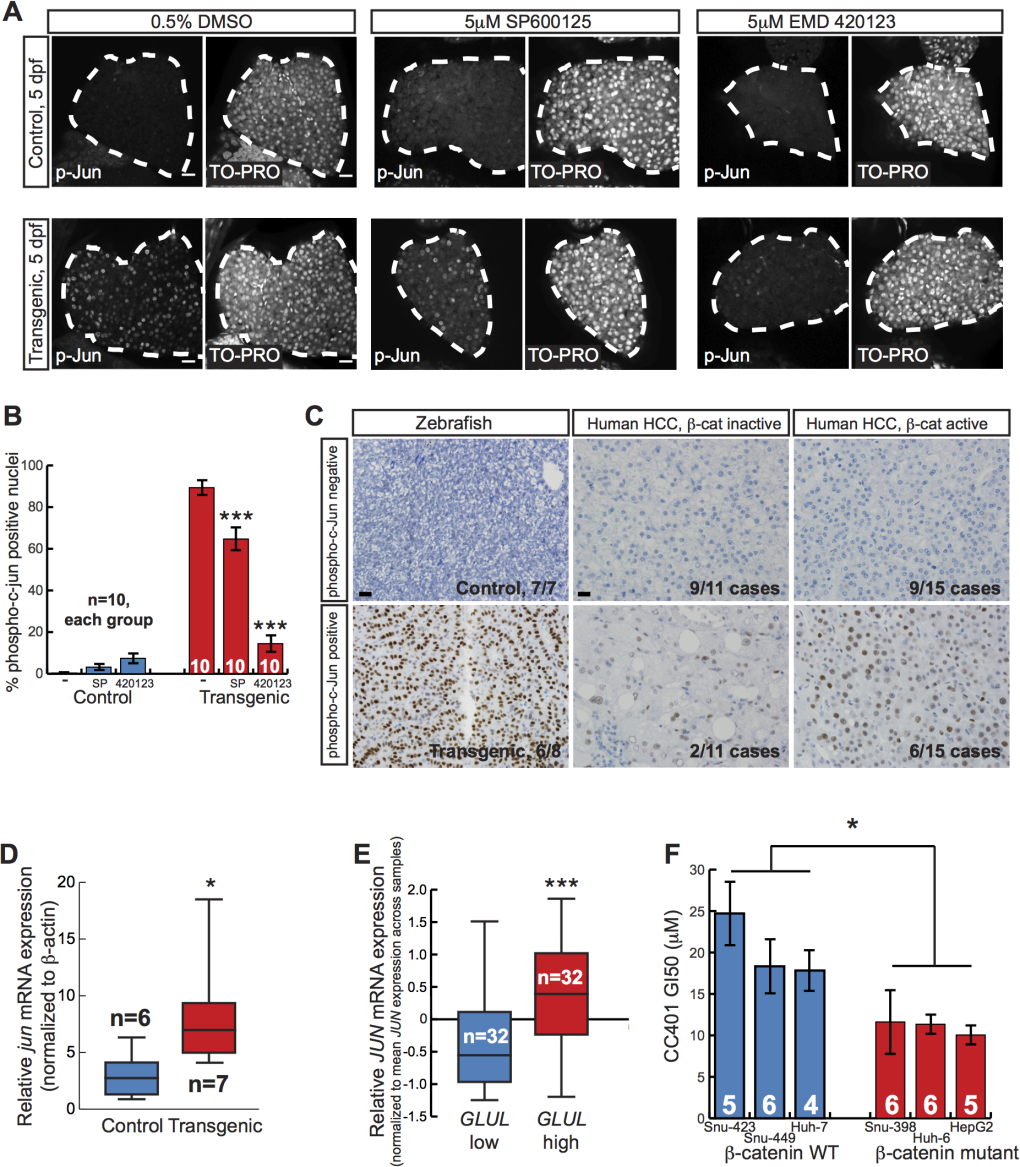
Activated β-catenin is associated with JNK pathway activation. (**A**) Representative whole-mount immunofluorescence images and **(B)** quantification of 5-day-old control sibling and transgenic larvae treated for 2 days with 0.5% DMSO alone, 5 μM SP600125, or 5 μM EMD 420123 and stained with antibodies against phospho-c-Jun (left panels) and TO-PRO nuclear stain (right panels). Livers are outlined in white. Scale bar, 20 μm. N values are shown above the x-axis. Drug-treated zebrafish were compared to DMSO-treated siblings with the same genotype using 2-way ANOVA. ***, p<0.001. (**C**) Representative photographs of zebrafish control and *Tg(fabp10a*:*pt-β-cat)* livers (left) and human HCC without (middle) and with (right) activated β-catenin, stained with anti-phospho-c-Jun antibodies. For zebrafish, 6 out of 8 (75%) *Tg(fabp10a*:*pt-β-cat)* livers with HCC contained one or more foci with moderate or high nuclear phospho-c-Jun staining (phospho-c-Jun positive), while all non-transgenic control livers (N = 7) without activated β-catenin showed absent or low staining (phospho-c-Jun negative). For human HCC, the number of cases exhibiting each staining pattern and the total number of cases with a given β-catenin activation status are shown at the bottom right of each picture. Images counterstained with hematoxylin. Scale bars, 20 μm. (**D**) Normalized *jun* mRNA expression in control sibling and transgenic adult zebrafish livers. Three technical replicates were performed for each sample. *, p<0.05, Mann-Whitney test. N values are shown above the x-axis. **(E)** Normalized *JUN* mRNA expression for human HCC with low *GLUL* and high *GLUL* expression. ***, p<0.001, Mann-Whitney test. (**F**) Graph showing dose of JNK inhibitor CC401 at which cell viability of human cancer cell lines was decreased by 50% (GI50), ± SEM. Number of replicates for each cell line is shown above the x-axis. *, p<0.05, unpaired t-test.

Our result that two JNK inhibitors decreased liver size in zebrafish expressing activated β-catenin raised the possibility that activated β-catenin might cooperate with the JNK pathway to increase liver size. Evidence from several different tumor models indicates that JUN is an important phosphorylation target of JNK during carcinogenesis[34]. Thus, we performed whole-mount immunofluorescence staining of zebrafish larvae using phospho-c-Jun-specific antibodies. *Tg(fabp10a*:*pt-β-cat)* zebrafish showed a higher index of nuclear phospho-c-Jun staining than controls, supporting the hypothesis that activated β-catenin is associated with JNK pathway activation in the zebrafish larval liver (Fig 5A and 5B). Furthermore, both JNK inhibitors significantly decreased phospho-c-Jun staining in transgenic larvae, consistent with the hypothesis that these compounds decrease liver size in *Tg(fabp10a*:*pt-β-cat)* zebrafish larvae at least in part by inhibiting the JNK pathway (Fig 5A and 5B). JNK inhibitor treatment did not significantly affect Wnt reporter activity or *Tg(fabp10a*:EGFP) expression in *Tg(fabp10a*:*pt-β-cat)* zebrafish larvae (S7 Fig), arguing that these drugs act downstream of or in parallel to β-catenin/TCF-driven transcription rather than by decreasing *fabp10a* promoter activity, altering transgene levels, and/or directly inhibiting β-catenin.

*JUN* autoregulates its own transcription via enhancer elements in its promoter[34,35] and forms a complex with β-catenin and TCF4 to activate *JUN* transcription in a JNK-dependent manner during mouse intestinal tumor formation[36]. Given our finding that activated β-catenin was associated with JNK pathway activation during zebrafish liver development, we hypothesized that a similar cooperation between activated β-catenin and the JNK pathway might occur during zebrafish liver tumor formation. To assess JNK pathway activity in adult zebrafish, we performed immunohistochemical staining using phospho-c-Jun-specific antibodies. We found that 6 out of 8 (75%) *Tg(fabp10a*:*pt-β-cat)* livers with HCC contained foci with moderate or strong nuclear phospho-c-Jun staining, while 0 out of 7 control livers without activated β-catenin showed such staining (Fig 5C; p<0.01, Fisher’s exact test). Furthermore, we found that *Tg(fabp10a*:*pt-β-cat)* livers had more *jun* expression than control livers (Fig 5D). Together, these results support the hypothesis that activated β-catenin is associated with JNK pathway activation in adult zebrafish livers as well.

### JNK activity in human HCC

Our chemical screen results and follow-up experiments indicate that activated β-catenin is associated with JNK pathway activation in zebrafish larvae and adult livers. We next sought to explore the relevance of this association to human liver tumorigenesis. Diffuse strong glutamine synthetase staining[37] and higher *GLUL* expression[5] are associated with β-catenin mutations, and these criteria can be used to identify β-catenin-activated tumors[38]. Therefore, we classified β-catenin activation status in human tumors based on glutamine synthetase staining pattern or *GLUL* expression levels. We found that 6 out of 15 (40%) of HCCs with activated β-catenin showed at least focal phospho-c-Jun staining (Fig 5C). We observed phospho-c-Jun staining less often in HCC without β-catenin activation (2 out of 11 tumors; 18%), although this difference was not statistically significant. Next, we investigated JNK activity in a separate, larger set of HCC specimens. Examining expression profiles of 96 HCC samples[39], we determined that tumors in the highest tertile of *GLUL* expression had significantly higher *JUN* levels than tumors in the lowest tertile (Fig 5E). These data suggest that JNK pathway activation is associated with activated β-catenin in human HCC.

Our finding in zebrafish larvae that JNK inhibition decreased liver size and increased survival specifically in the context of activated β-catenin raises the possibility that β-catenin activation might confer sensitivity to JNK inhibition in other systems, including human liver cancer. To test this hypothesis, we used a panel of human liver tumor cell lines with and without activating mutations in β-catenin[40,41], treating cells with the newer generation JNK inhibitor CC401 that is currently in clinical trials. The concentration of CC401 that inhibited cell growth by 50% (GI50) was significantly lower in cells with activating mutations in β-catenin than in cell lines without β-catenin mutations, indicating an increased sensitivity to this drug (Fig 5F) (p < 0.05, unpaired t test).

### Antidepressants suppress β-catenin-induced liver enlargement

**Fig 6:**
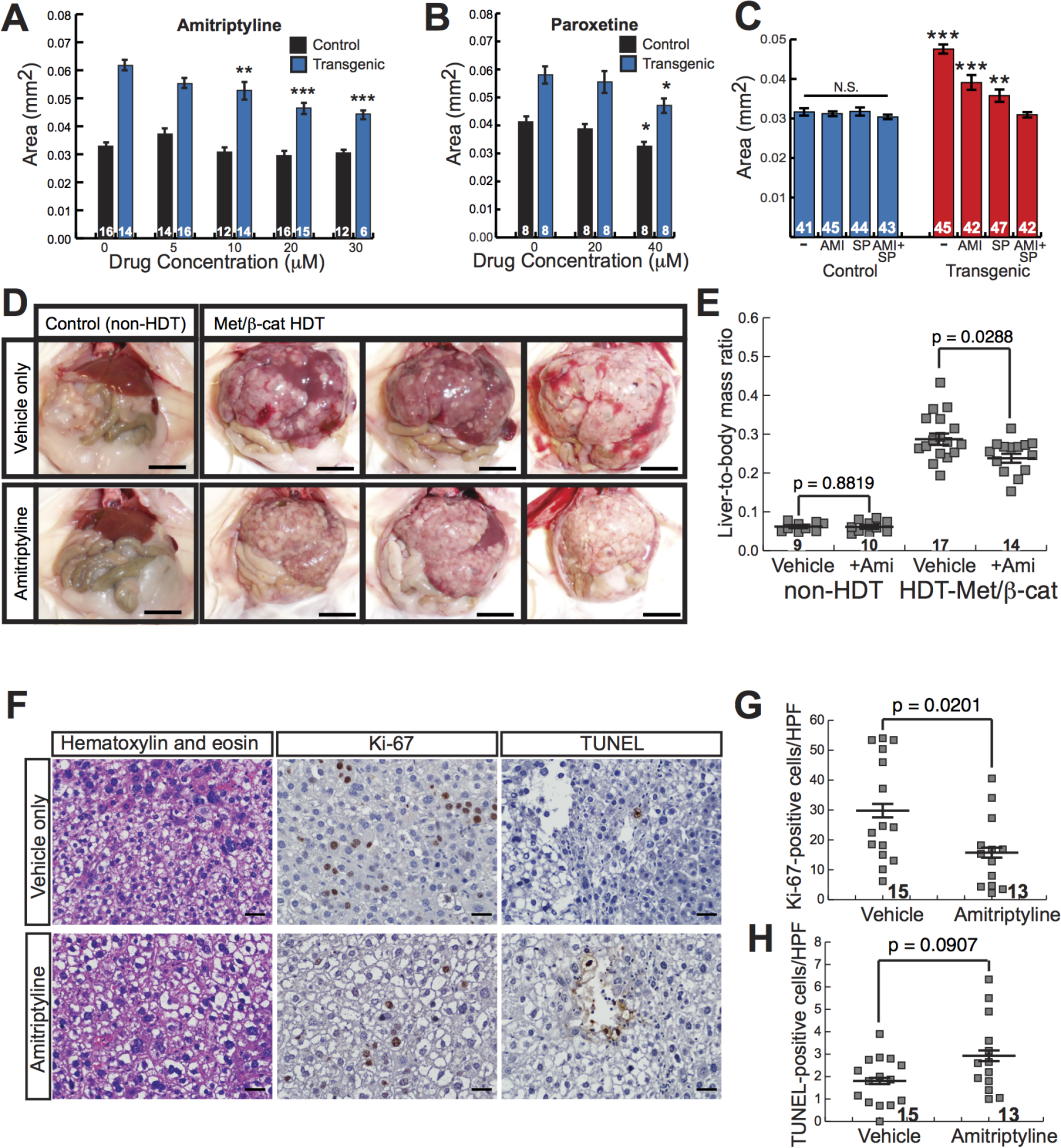
Antidepressants decrease β-catenin-induced liver enlargement and tumorigenesis. (**A-B**) Graphs showing mean liver size ± SEM of 6-day-old control sibling and transgenic zebrafish larvae treated for 3 days with amitriptyline (**A**) or paroxetine (**B**) at the indicated dosages. N values are shown above the x-axis. Asterisks indicate p-values for 2-way ANOVA comparing drug-treated zebrafish to DMSO-treated siblings with the same genotype: *, p<0.05; **, p<0.01; ***, p<0.001. (**C**) Graph showing mean liver size ± SEM of 6-day-old control sibling and transgenic zebrafish larvae treated for 3 days with 0.5% DMSO (-), 20 μM amitriptyline (AMI), 2 μM SP600125 (SP), or both drugs combined (AMI+SP). N values are shown above the x-axis. Asterisks indicate p-values for 2-way ANOVA comparing each group of transgenic zebrafish to AMI+SP group: **, p<0.01; ***, p<0.001. N.S., no significant difference between groups of non-transgenic zebrafish (2-way ANOVA). (**D**) Representative images of control, non-hydrodynamically transfected (non-HDT) mouse livers (left) and mouse liver tumors induced by hydrodynamic transfection of activated β-catenin and Met (Met/β-cat HDT). Mice were treated with saccharine alone (vehicle only, top row) or amitriptyline plus saccharine (bottom row). Scale bars, 1 cm. (**E**) Graph showing mean liver-to-body mass ratios ± SEM for non-HDT and HDT-Met/β-cat mice treated with saccharine alone (vehicle) or amitriptyline plus saccharine (+Ami). P values calculated with Mann-Whitney test. N values are shown above the x-axis. (**F**) Representative hematoxylin-and-eosin-stained, Ki-67-labeled, and TUNEL-labeled images from vehicle- and amitriptyline-treated mice. Ki-67 and TUNEL stainings were performed using 3, 3'-diaminobenzidine (DAB) substrate, so positive-staining cells are brown, and hematoxylin counterstain to highlight nuclei and other basophilic structures in blue. (**G-H**) Graphs showing mean ± SEM of Ki-67-positive (**G**) or TUNEL-positive (**H**) cells per high-power field. P values calculated with Mann-Whitney test. N values are shown above the x-axis.

Having established our zebrafish model as a useful tool for identifying drugs that suppress β-catenin-induced liver tumorigenesis, we next expanded our chemical screen to search for additional, novel compounds with anti-HCC effects. We tested 800 compounds from a commercially available library of pharmacologically active compounds, many of which are FDA-approved for treatment of non-cancer illnesses, reasoning that hits from such a screen could more easily be repurposed for HCC treatment. We identified two drugs, amitriptyline (a TCA) and paroxetine (an SSRI) that decreased liver size in *Tg(fabp10a*:*pt-β-cat)* zebrafish without causing obvious toxicity (S5 Table). Both antidepressants caused a dose-dependent decrease in liver size in *Tg(fabp10a*:*pt-β-cat)* larvae (Fig 6A and 6B). As we found with SP600125, amitriptyline treatment did not significantly affect Wnt reporter activity or *Tg(fabp10a*:EGFP) expression in *Tg(fabp10a*:*pt-β-cat)* zebrafish larvae (S7 Fig), arguing that this drug acts downstream of or in parallel to β-catenin/TCF-driven transcription rather than by decreasing *fabp10a* promoter activity, altering transgene levels, and/or directly inhibiting β-catenin.

To identify additional related compounds that suppress β-catenin-induced larval liver enlargement, we screened 80 compounds from a serotonergic ligands library. We found two additional drugs, mirtazapine (a tetracyclic antidepressant) and clomipramine (a TCA), that decreased liver size in *Tg(fabp10a*:*pt-β-cat)* zebrafish without causing obvious toxicity (S5 Table). The identification of four antidepressants, all of which inhibit serotonin reuptake, that suppress β-catenin-mediated liver growth raises the possibility that serotonin signaling or another shared target of these drugs may inhibit β-catenin-induced liver tumorigenesis.

Combining multiple drugs in cancer treatment represents an important tool for achieving maximal therapeutic benefit. To investigate the possibility that JNK inhibitors and serotonergic antidepressants could be used together to decrease β-catenin-mediated liver growth, we examined the effects of SP600125 plus amitriptyline on *Tg(fabp10a*:*pt-β-cat)* larval liver size. Treatment with high doses of both drugs (5 μM SP600125 plus 30 μM amitriptyline) resulted in death in 20 out of 20 larvae treated with drugs from 3 dpf to 6 dpf. However, treatment with slightly lower doses of both drugs (2 μM SP600125 plus 20 μM amitriptyline) resulted in a decrease in liver size that was significantly greater than the one we observed with either drug alone (p<0.001 versus 20 μM amitriptyline and p<0.01 versus 2 μM SP600125, 2-way ANOVA)(Fig 6C). This drug combination did not significantly alter liver size in non-transgenic animals (2-way ANOVA)(Fig 6C). Livers of *Tg(fabp10a*:*pt-β-cat)* zebrafish treated with the two-drug combination were similar in size to livers of non-transgenic siblings (Fig 6C). These data suggest that amitriptyline and SP600125 decrease liver size in *Tg(fabp10a*:*pt-β-cat)* zebrafish via mechanisms that are at least partially distinct.

We next sought to test the hypothesis that amitriptyline, which caused the most robust decrease in liver size in *Tg(fabp10a*:*pt-β-cat)* zebrafish among the antidepressants we tested, had an anti-tumor effect in human liver cancer cells. To test this hypothesis, we treated a panel of human liver tumor cell lines with and without activating mutations in β-catenin[40,41], as well as immortalized non-transformed human hepatocytes, with amitriptyline. Interestingly, every human liver cancer cell line had a GI50 that was at least slightly lower than the non-tumor HepTert cell line, indicating an increased sensitivity to this drug (S8 Fig). While on average cell lines with activating mutations in β-catenin had a lower GI50 (17.7 μM) than cell lines without β-catenin mutations (21.7 μM), this difference was not statistically significant, raising the possibility that other genetic or epigenetic differences besides β-catenin mutation might contribute to amitriptyline sensitivity in liver cancer cells.

In contrast to our other class of hits, the JNK inhibitors, antidepressants are FDA approved and are widely used for treatment of a variety of common, non-neoplastic conditions including major depressive disorder, obsessive-compulsive disorder, and anxiety disorders. In addition, these compounds are highly soluble in water and easy to administer. We reasoned that together these attributes could facilitate translation of our amitriptyline studies to other vertebrates, including mammals. Although β-catenin alone is not sufficient to induce HCC in mice, hydrodynamic transfection of *MET* and activated β-catenin together (HDT-Met/β-Cat) induces multi-focal HCC in mice[42], resulting in innumerable (greater than 100) coalescing tumors that replace the majority of the hepatic parenchyma (Fig 6D, top panels, and S9A–S9B Fig top panels). Therefore, we next sought to test the hypothesis that amitriptyline could decrease tumorigenesis in HDT-Met/β-Cat mice. We treated these mice continuously with 200 mg/L amitriptyline in their drinking water, to achieve plasma levels of drug that improve behavior in mouse models of depression[43,44] and are similar to the therapeutic levels in humans treated for depression[43].

Strikingly, we found that amitriptyline treatment caused a significant decrease in the liver tumor burden of HDT-Met/β-Cat mice, decreasing mean liver-to-body mass ratio from 0.29 to 0.24 (p = 0.0288) (Fig 6D and 6E). Amitriptyline treatment decreased mean liver mass from 6.8 g to 4.9 g (p = 0.0012) and decreased mean body mass from 23.0 g to 20.1 g (p = 0.0023) in HDT-Met/β-Cat mice (S10 Fig). In mice that had not been hydrodynamically transfected, amitriptyline treatment did not significantly change liver-to-body mass ratio (mean = 0.061 in vehicle- and amitriptyline-treated mice; p = 0.8819) or liver mass (mean = 1.7 g in vehicle-treated mice; mean = 1.6 g in amitriptyline-treated mice; p = 0.6724), although in these mice amitriptyline caused a slight decrease in body mass (mean = 27.3 g in vehicle-treated mice; mean = 26.0 g in amitriptyline-treated mice) that was not statistically significant (p = 0.0749) (Figs 6D, 6E and S10). Upon histologic examination of HDT-Met/β-cat livers, we noted that amitriptyline treatment did not substantially alter tumor morphology (S9A–S9B Fig bottom panels). However, amitriptyline treatment significantly decreased tumor cell proliferation (Figs 6F, 6G, and S11), reducing the amount of Ki-67 labeling from 30 to 16 Ki-67-positive cells per high-power field (HPF) (p = 0.0201). At the same time, amitriptyline-treated tumors demonstrated slightly increased cell death, with 2.9 TUNEL-positive cells per HPF compared to 1.8 TUNEL-positive cells per HPF in control tumors, although this difference was not statistically significant (p = 0.0907) (Figs 6F, 6H, and S11). Together, these findings indicate that amitriptyline, in addition to decreasing β-catenin-induced liver enlargement in zebrafish, inhibits liver tumorigenesis in a mammalian model.

## Discussion

In this study, we report a new transgenic zebrafish model with hepatocyte-specific activation of β-catenin. We found that activation of β-catenin promotes liver overgrowth in zebrafish, consistent with mouse models where its activation leads to increased hepatocyte proliferation and liver growth[13]. While β-catenin activation alone does not give rise to HCC in mouse models[6,13], our study reveals that it is sufficient to promote HCC in adult zebrafish. Considering that β-catenin activation may be an early or initiating event in some human HCC[4,7,8], our new zebrafish transgenic line represents a unique and useful tool to study mechanisms of β-catenin-mediated liver tumorigenesis and identify potential therapeutics for these tumors.

Using these transgenic zebrafish, we performed a small-molecule screen and identified several kinase inhibitors that decreased liver size in larvae expressing hepatocyte-specific activated β-catenin. We focused our follow-up studies on two JNK inhibitors, and found that expression of activated β-catenin was associated with JNK pathway hyperactivation in larval and adult livers. Prompted by our observations in zebrafish, we examined human tumors for evidence of JNK pathway activity and noted increased *JUN* expression predominantly in HCCs with activated β-catenin, demonstrating the link between the two in human disease. Although previous studies had reported a role for JNK in liver tumorigenesis[45], a link between JNK activation and a specific HCC subtype had not been previously established. Our studies raise the possibility that JNK activity may be of particular importance in HCC with activated β-catenin.

Phospho-c-Jun staining in HCC is associated with poor response to sorafenib[46], the only drug currently approved for treatment of advanced HCC[1], underscoring the importance of identifying more effective therapies for HCCs with JNK activation. Our results connecting activated β-catenin and the JNK pathway raise the possibility that sorafenib resistance may be linked to activating mutations in β-catenin in human HCC. We propose that inhibiting the JNK pathway may represent an especially useful therapeutic strategy for this sorafenib-resistant HCC subgroup. SP600125 suppresses liver tumor formation in *N*-diethylnitrosamine (DEN) treated rats[47], indicating its anti-tumor potency in mammals. However, as the tumors in that study were induced by a chemical carcinogen and not by a specific genetic manipulation, the relevance of these findings to any of the genetically distinct human HCC subtypes was not clear. Now, our findings provide the first evidence that SP600125 or related compounds might be effective in treating human HCC with β-catenin mutations.

Repurposing existing FDA-approved drugs for cancer treatment has several advantages, including decreased drug development time. The need for new systemic therapies is particularly urgent for HCC, given its increasing incidence and its high mortality rate when localized treatment fails. In the second round of our chemical screen, we tested compounds with known mechanisms of action, including many FDA-approved drugs, and identified two antidepressants, a TCA and an SSRI, that suppressed β-catenin-induced liver growth in our zebrafish model. We also found that one of these drugs, amitriptyline, decreased tumor burden in a mouse HCC model. Our finding that these antidepressants decreased liver growth in these tumor models was somewhat surprising, because both TCAs and SSRIs are thought to act at least in part by increasing serotonin levels in the brain via inhibition of the serotonin reuptake transporter (SERT), and serotonin promotes liver growth during regeneration[48]. However, TCAs and SSRIs have other biologically important roles besides SERT inhibition, including direct and/or indirect effects on serotonin receptors (5-HT2A and 5-HT2B)[49,50]. In addition to blocking both serotonin and norepinephrine reuptake, TCAs bind to multiple G-protein-coupled cell surface receptors, mediating induction of apoptosis in lung cancer cells[51]. Furthermore, it is possible that in the models we used, TCAs and SSRIs are exerting at least part of their anti-tumor effects via non-tumor cell types, such as hepatic stellate cells, which store, release, and respond to serotonin[52]. Here we show that amitriptyline inhibited β-catenin-induced liver growth and/or tumorigenesis using two distinct, *in vivo*, vertebrate systems. Further experiments are required to determine which cell types and molecular targets mediate the anti-tumor effects of amitriptyline in these models. Although the myriad targets of antidepressants complicate mechanistic investigations, the pleiotropic effects of these drugs may render them particularly suitable for repositioning as cancer therapeutics. Our findings raise the exciting possibility that amitriptyline or related antidepressants could be effective in treating or preventing a major subset of human HCC.

Although we found that liver cancer cell lines with β-catenin mutations had a slightly lower GI50 in response to amitriptyline than cell lines without β-catenin mutations, this difference was not statistically significant, and we noted that 5 out of 6 liver cancer cell lines had a significantly lower GI50 in response to amitriptyline than immortalized hepatocytes. These results raise the possibility that amitriptyline might be generally toxic to cancer cells, as opposed to having an effect that is specific to the presence of activated β-catenin. In keeping with this possibility, previous reports have shown that amitriptyline inhibits multiple cancer types *in vitro*, including liver cancer[53], multiple myeloma[54], melanoma[55], small cell lung cancer[51], glioma[56], and neuroblastoma[56]. Amitriptyline has direct actions on tumor cells, such as inducing mitochondrial damage, increasing reactive oxygen species, and inhibiting the antioxidant machinery of human cervical, lung, and liver cancer cells[57]. We discovered that amitriptyline caused a significant decrease in liver tumor burden in mice hydrodynamically transfected with Met and β-catenin, but we did not test the effects of amitriptyline on mouse liver tumors lacking activated β-catenin. Future studies examining the effects of amitriptyline in other mouse models could be helpful to determine the specificity or generalizability of this drug’s anti-tumor effects.

Previous chemical screens for inhibitors of Wnt/β-catenin signaling have primarily employed cell-based assays[58,59]. These approaches are relatively fast, enabling testing of very large numbers of compounds. While we were able to test only a modest number of compounds in our chemical screen, the whole organism approach facilitates examination of organ-specific phenotypes and enables the identification of compounds that may more indirectly affect Wnt/β-catenin signaling, for example via downstream targets, parallel cooperating pathways, or effects on non-hepatocyte cell types. Furthermore, our approach enabled us to concomitantly assess toxicity in live vertebrate animals.

During our study, we focused on mechanistic characterization of two compounds identified in our chemical screen because these drugs shared a single well-described mechanism of action, JNK inhibition. Our subsequent discovery that JNK activity is relevant to both the larval and adult phenotypes of our model as well as to human HCC validates this chemical screen as a productive approach to identify and explore additional mechanisms translatable to human β-catenin-mediated liver tumorigenesis.

## Materials and Methods

### Zebrafish strains and generation of *Tg(fabp10a*:*pt-β-cat)* zebrafish

Male and female wild-type AB and TL, *Tg(7xTCF-Xla*.*Siam*:*GFP)* ^*ia4*^[21], *Tg(-2*.*8fabp10a*:*EGFP)* ^*as3*^[17] and *Tg(fabp10*:*rasGFP)* ^*s942*^[60] strains were maintained as described[61].

We amplified *pt-β-catenin* by polymerase chain reaction (PCR) from the pCS2-XE49 plasmid[16], a kind gift from Ben Cheyette, adding NotI and ClaI restriction sites at the 5’ and 3’ ends, respectively. We placed the *pt-β-catenin* insert between the ~2.8 kB *fabp10a* upstream region[17] and polyadenylation sequences into a vector containing, in reverse orientation, the *crystallin alpha A* eye specific promoter (*cryaa)* followed by coding sequence for Venus fluorescent protein[19,20]. The resulting plasmid also contained I-SceI restriction enzyme sites, facilitating generation of transgenic zebrafish as previously described[62]. One-cell stage embryos were microinjected with plasmid and I-SceI enzyme (New England Biolabs, Ipswich, MA) using a Leica dissecting microscope (Allendale, NJ). Injected embryos with yellow-green eyes at 2–3 dpf were raised to adulthood. By crossing these injected animals to wild-type zebrafish, we identified three founders representing independent transgenic lines 3–3 (*s985*), 4–1 (*s986*), and 4–5 (*s987*). Transgenic descendants of these founders were distinguished from control siblings based on *cryaa*:Venus expression at 3 dpf or later.

### Analysis of *Tg(fabp10a*:*pt-β-cat)* adult liver morphology

For analysis of gross and microscopic changes in adult zebrafish over time, we raised and maintained 5 clutches of *Tg(fabp10a*:*pt-β-cat)* ^*s987*^ zebrafish and parallel tanks of non-transgenic control siblings. At each time point, zebrafish were randomly selected from the *Tg(fabp10a*:*pt-β-cat)* tank and the control tank containing zebrafish from the same clutch or clutches. Zebrafish were euthanized at the following time points by rapid chilling and weighed: 2 months (6 control zebrafish, 6 transgenic zebrafish); 3 months (8 control zebrafish, 8 transgenic zebrafish); 4 months (10 control zebrafish, 15 transgenic zebrafish); 5 months (8 control zebrafish, 8 transgenic zebrafish); 6 months (9 control zebrafish, 9 transgenic zebrafish); 12 months (10 control zebrafish, 10 transgenic zebrafish). Livers were dissected using a Leica dissecting microscope, rinsed in phosphate buffered saline (PBS), and weighed, and the entire liver or representative portions were fixed in 10% formalin for at least 24 hours. Remaining liver portions were used for RNA preparation and/or cryosections as described below. Paraffin embedding, sectioning, and hematoxylin and eosin (H&E) staining were performed at the Gladstone Histology and Light Microscopy Core Facility. Slides were blinded and histologic changes were scored by a pathologist (K.J.E.). A second pathologist (J.S.) reviewed a subset of the slides to confirm the diagnoses. Each specimen was assigned to one of the following categories: (1) Hepatocellular carcinoma (HCC), defined as the presence of architectural abnormalities, such as pseudoglands, sheets of cells, and decreased or absent bile ducts, involving greater than 5% of the tissue, plus cytological abnormalities, including nuclear enlargement, nuclear contour irregularities, and mitotic figures; (2) Minimal/mild changes, defined as the presence of cytological abnormalities in the absence of substantial architectural disruption or architectural abnormalities without substantial cytological atypia; or (3) No changes, defined as no substantial cytological or architectural abnormalities. Lesions assigned to the first category (HCC) fulfilled diagnostic criteria established by a board-certified pathologist with subspecialty fellowship training in hepatopathology (K.J.E.) (S2 Table) as well as by other pathologists and veterinary pathologists [22]. Quantification of liver-to-body mass ratios and microscopic changes over time were performed in the same experiment. In some cases, only a subset of zebrafish were examined microscopically, so N values are lower for microscopic analysis of the following groups: transgenic zebrafish at 4 months (N = 8); control zebrafish at 3, 4, and 5 months (N = 4, each time point).

Representative photographs of livers situated within the body cavities of 4-month-old *Tg(fabp10a*:*pt-β-cat)* ^*s986*^ zebrafish and control non-transgenic siblings were taken with a Zeiss Stemi SV 11 microscope and Zeiss MRc5 camera (Carl Zeiss, Oberkochen, Germany) at lowest magnification. Representative photographs of adult zebrafish and human liver sections were taken with an Olympus BX51 microscope using the 40X objective and an Olympus DP72 camera (Olympus, Tokyo, Japan).

### Flow cytometric analysis

Two separate independent flow cytometry experiments were performed, which showed similar results. For experiment #1, we harvested cells from 4-month-old *Tg(fabp10a*:*pt-β-cat)* ^*s987*^ zebrafish and control siblings by dissecting out livers, dissociating cells in 0.25% trypsin, and filtering cells through a 40 µm filter. The concentration of live cells in each sample was determined using a Countess Automated Cell Counter (Life Technologies, Carlsbad, CA, USA), in order to prepare 500 µL cell suspensions with equal numbers of live cells. Cell were fixed overnight in 95% ethanol at -20ºC and then stained with propidium iodide in the presence of RNase A for 1 hour at room temperature. Flow cytometry was performed using a BD LSRII with FACSDiva Software (BD, Franklin Lakes, NJ); 5,500 to 30,000 events were collected for each sample. For experiment #2, we harvested, fixed, and stained cells from 4-month-old *Tg(fabp10a*:*pt-β-cat)* ^*s985*^ zebrafish, 18-month-old *Tg(fabp10a*:*pt-β-cat)* ^*s987*^ zebrafish, and 18-month-old control siblings using the same protocol as for experiment #1. Flow cytometry was performed using a BD Fortessa with FACSDiva Software (BD); 10,000 events were collected for each sample. We then spiked each liver sample with a few drops of fixed, propidium-iodide-stained sperm obtained from non-transgenic adult zebrafish in order to provide a 1N reference; 10,000 additional events were collected for each sample. We visually confirmed that plots for samples with and without sperm appeared similar except for the presence of a 1N peak.

Raw data collected from both experiments were gated sequentially as follows: first, dead and dying cells were excluded by plotting side-scatter area (SSC-A)(y-axis) versus forward-scatter area (FSC-A)(x-axis) and gating out cells on the far left; second, doublets were excluded by plotting forward-scatter width (FSC-W) versus FSC-A and gating out cells with high FSC-W; third, remaining doublets were excluded by plotting side-scatter width (SSC-W) versus SSC-A and gating out cells with high SSC-W. Histograms showing live, doublet-free cells were generated using FACSDiva Software.

### Microarray analysis

We isolated RNA from 4-month-old *Tg(fabp10a*:*pt-β-cat)* ^*s986*^ zebrafish livers with HCC and control sibling livers. Preparation of cDNA and subsequent microarray hybridizations using Agilent zebrafish (V3) microarrays were performed at MoGene (St. Louis, MO). Two biological replicates were performed, and the fold-change for each probe was determined by dividing the processed Cy3 (transgenic) signal by the processed Cy5 (control) signal. The average fold-changes of probes with significant signals above background were inputted into Ingenuity Pathways Analysis (IPA) (Ingenuity Systems, www.ingenuity.com, release date 2012-11-01).

### Zebrafish RNA-seq analysis, human microarray analysis, and cross-species comparison, hierarchical clustering, and pathway enrichment analyses

We isolated RNA from 4- to 6-month-old *Tg(fabp10a*:*pt-β-cat)* ^*s986*^ and *Tg(fabp10a*:*pt-β-cat)* ^*s987*^ zebrafish livers with HCC and control sibling livers. Bioanalyzer trace and RNA-seq library preparation were performed at the Gladstone Genomics Core Laboratory. The UCSF Genomics Core Facility performed single-read, 50-base-pair sequencing using Illumina HiSeq technology; 8 pooled barcoded libraries were run in one lane of the flow cell. Raw FASTQ files, corresponding to each sample, were aligned to Release 79 of the zebrafish genome made available by the Ensembl project, using STAR v2.4[63]. Overlaps of the resulting alignments with protein-coding genes were then quantified using the GenomicAlignments R package[64]. Differential gene expression analysis between *Tg(fabp10a*:*pt-β-cat)* animals and their wild-type siblings was performed using the DESeq2 R package[65]. Regularized log-transformed values of the raw counts were used for all subsequent cross-species analyses.

Gene expression profiles for 268 human HCC tumors and 243 adjacent non-tumor liver tissue, measured using a Rosetta/Merch RSTA Affymetrix microarray, were sourced from the Gene Expression Omnibus (GEO–Accession: GSE25097)[30–32]. Probes corresponding to multiple Entrez IDs were collapsed using the ‘MaxMean’ method in the WGCNA R package[66–68]. Differential gene expression analysis between HCC samples and the adjacent non-tumor liver tissue was then performed on log2-transformed values using the limma R package[69].

To enable cross-species comparison, zebrafish genes were matched to their human orthologs based on annotations available from the Ensembl database, using the biomaRt R package[70,71]. One-to-many relationships were resolved by choosing the orthologous pairs with the largest confidence of orthology and amino acid sequence identity with respect to the human protein, as indicated in the Ensembl annotations. A total of 13710 orthologous pairs with one-to-one relationships between zebrafish and human genes were obtained as such.

For cross-species clustering analysis, human and zebrafish gene expression values were first individually median-centered and then scaled by dividing each value by the median absolute deviation for the corresponding gene. The transformed datasets were then merged and subjected to principal component analysis (PCA) and hierarchical clustering using a Pearson correlation distance metric with average linkage, based on a subset of 283 orthologous gene pairs that are significantly dys-regulated in human HCC samples when compared to adjacent non-tumor liver samples (absolute log fold-change ≥ 2, false discovery rate (FDR) < 0.05). PCA and hierarchical clustering were performed using the FactoMineR[72] and cluster[73] R packages respectively.

To probe for conserved genetic pathways, we focused on genes that were significantly dys-regulated in the same direction in both human HCC samples and *Tg(fabp10a*:*pt-β-cat)* animals when compared to their non-tumor counterparts (FDR < 0.05). Expression profiles of these genes are illustrated in heatmaps for human and zebrafish samples respectively, using the gplots[74] and cluster[73] R packages. We then performed pathway enrichment analysis on this gene list. Overrepresented Gene Ontology (GO) terms [75] and pathways from the Kyoto Encyclopedia of Genes and Genomes (KEGG) [76] and Reactome [77] databases were identified using a p-value cutoff of 0.05 as determined by the Fisher’s Exact Test.

### Chemical screen and follow-up

We used the InhibitorSelect 384-Well Protein Kinase Inhibitor Library I (EMD Millipore/Calbiochem, Billerica, MA), the Library of Pharmacologically Active Compounds (LOPAC)(Sigma-Aldrich, St. Louis, MO), and the Screen-Well Serotonergic ligand library (Enzo Life Sciences, Farmingdale, NY), supplied as concentrated DMSO solutions. Compounds or DMSO control were dissolved into separate wells of a 96-well glass bottom microwell plate (Matrical Bioscience, Spokane, WA) to a final volume of 500 µL and final concentration of 2 μM and 0.2% DMSO (kinase inhibitor library) or 10 0μM and 1% DMSO (other libraries). In the initial screen, for each compound we tested at least 2 wells containing *Tg(fabp10a*:*pt-β-cat); Tg(fabp10a*:*EGFP)* larvae and at least one well containing *Tg(fabp10a*:*EGFP)* control siblings. Three larvae were placed into each well. Larvae were sorted under a Leica MZ16F fluorescence dissecting microscope based on *cryaa*:Venus and *fabp10a*:EGFP expression at 3 dpf, pipetted into wells, cultured at 28°C, and examined 3 days later at 6 dpf using the same microscope. We scored liver size qualitatively on a scale from 0 to 4, with 1 indicating typical liver size in 6 dpf *Tg(fabp10a*:*EGFP)* larvae without activated β-catenin in the absence of drug, 0.5 indicating smaller-than-typical liver size, and 2–4 indicating increased liver size. For each plate, we included at least 4 control wells containing *Tg(fabp10a*:*pt-β-cat); Tg(fabp10a*:*EGFP)* larvae in DMSO, and experimental compounds were only considered potential hits if the average liver size score in these control wells was greater than or equal to 2. Drugs that decreased average liver size of *Tg(fabp10a*:*pt-β-cat); Tg(fabp10a*:*EGFP)* zebrafish in both wells (average liver size score less than 2) without causing toxicity/death in any wells were considered potential hit compounds.

The secondary validation screen was performed using a similar protocol, except for every drug we tested at least three wells, each containing three larvae. Drugs that resulted in an average liver size score of less than or equal to 2 in *Tg(fabp10a*:*pt-β-cat); Tg(fabp10a*:*EGFP)* zebrafish without causing toxicity/death in control *Tg(fabp10a*:*EGFP)* siblings were considered confirmed hits. Again, compounds were only considered hits if the average liver size score in the corresponding *Tg(fabp10a*:*pt-β-cat); Tg(fabp10a*:*EGFP)* DMSO control well(s) was greater than or equal to 2.

Additional follow-up experiments were performed with SP600125 (Sigma-Aldrich), EMD 420123 (EMD Millipore/Calbiochem), amitriptyline (Sigma-Aldrich) and paroxetine (Sigma-Aldrich). We dissolved these compounds in DMSO and stored them as concentrated solutions at -20°C or -80°C. Larvae were cultured in egg water (40 g/L Instant Ocean) with drugs at a final concentration of 0.5–100 μM from 3 dpf until euthanasia and fixation; controls were cultured with the same DMSO concentration (0.2–1%).

### Analysis of *Tg(fabp10a*:*pt-β-cat)* survival

To generate adult survival curves, we raised and maintained 4 clutches of *Tg(fabp10a*:*pt-β-cat)* ^*s986*^ zebrafish and parallel tanks of non-transgenic control siblings. Beginning at adulthood (2–4 months post fertilization), zebrafish were counted and/or checked 2–3 times per week and the number of dead and missing zebrafish was recorded. Zebrafish with evidence of distress including altered swimming or feeding behavior or severe abdominal distention were euthanized and counted as dead. The experiment was terminated when all zebrafish were greater than 11 months old. Survival curves were generated and analyzed using MedCalc statistical software.

### Larval liver size measurements, immunofluorescence, and EdU labeling

In general, experiments involving zebrafish larvae were planned to result in a final sample size of approximately 7–10 zebrafish. With respect to randomization, animals were sorted at 3 dpf (before increased liver size of transgenic animals is obvious) and assigned to experimental groups immediately after sorting. Animals were excluded from analysis if they died during drug treatment (10 μM and 50 μM EMD 420123) or if livers became detached or poorly oriented during fixing, staining, or mounting. Exclusion of samples was established before quantification of results.

Larvae were euthanized with tricaine methanesulfonate (0.03%), fixed in 4% paraformaldehyde (PFA) in PBS for at least 12 hours at 4°C and rinsed in PBS. Skin and any residual yolk surrounding the liver were removed using forceps under a Leica dissecting microscope. Brightfield photographs of larvae embedded in 3% methylcellulose were taken using a Zeiss Stemi SV 11 microscope and Zeiss MRc5 camera at highest magnification. We quantified larval liver size in transgenic zebrafish (Tg1 = *s985*; Tg2 = *s986*; Tg3 = *s987*) and control siblings by outlining livers on these photographs and measuring the area using FIJI/ImageJ. For dose-response experiments, photographs were blinded before measuring liver size. Dose response experiments for each drug and drug combination were done at least twice on separate clutches of zebrafish, and showed similar trends; results shown are pooled.

Immunofluorescence microscopy was performed essentially as previously described[78]. Whole, partially dissected larvae were blocked with 4% bovine serum albumin (BSA) plus 0.3% Triton X-100 in PBS (PBT) for 2 hours at room temperature (for phospho-c-Jun-specific antibody staining) or for 1 hour at 4°C (all other primary antibodies). Larvae were incubated with one or more of the following primary antibodies diluted in PBT at 4°C for at least 16 hours: chicken antibody to green fluorescent protein (GFP) (Aves Labs, Tigard, Oregon; Catalog Number GFP-1010; 1:500 dilution); rabbit antibody to phospho-c-Jun Ser73 (Cell Signaling Technology, Beverly, MA; Catalog Number 9164; 1:100 dilution, pre-incubated with ~20 fixed larvae overnight before use); rabbit antibody to phospho-histone H3 (PHH3) (EMD Millipore; Catalog Number 06–570; 1:500 dilution) and mouse antibody to β-catenin (Sigma-Aldrich; Clone 15B8, Catalog Number C-7207; 1:200 dilution). Larvae were rinsed in PBS plus 0.3% Triton X-100 (PT) for 1–2 hours, incubated with appropriate corresponding Alexa Fluor secondary antibodies (Life Technologies) for at least 7 hours at 4°C, rinsed, and mounted in 1% agarose plus Vectashield Hardset Mounting Medium (Vector Labs, Burlingame, CA).

For studies of hepatocyte proliferation, larvae were incubated in 10 μM 5-ethynyl-2’-deoxyuridine (EdU) dissolved in embryo medium for 3 hours immediately before euthanasia. EdU was visualized using the Click-iT EdU Imaging Kit (Life Technologies), with the Click-iT reaction performed before incubation with primary antibodies.

Samples were imaged on a Zeiss LSM5 Pascal confocal microscope (Carl Zeiss) or a Nikon Eclipse 90i confocal microscope (Nikon Inc., Melville, NY).

### Quantification of hepatocyte proliferation, cell size, Wnt reporter activity, and phospho-c-Jun immunofluorescence staining in zebrafish larvae

To quantify hepatocyte proliferation and cell size, we used *Tg(fabp10a*:*pt-β-cat)* ^*s987*^ *; Tg(fabp10a*:*rasGFP)* larvae and *Tg(fabp10a*:*rasGFP)* control siblings. Larvae were fixed at 6 dpf, and proliferating hepatocytes and cell membranes were visualized by EdU labeling and anti-GFP immunofluorescence staining, respectively. Larvae were blinded and imaged using the same pinhole, laser power, slice number (50, beginning at ventral surface of liver), slice interval (1 μM), and scan speed for each larva, but gain was adjusted to obtain the clearest image. Images were blinded again before quantifying EdU positivity, which was determined by counting the number of EdU positive hepatocytes and the total number of hepatocytes in slice 8 using ImageJ. To quantify average cell size, we used the same set of blinded images. For each larva, twenty representative cells in slice 2 were outlined and measured using ImageJ.

The EdU and cell size experiments were done twice, and only representative results (experiment 2) are shown in Fig 2. We observed an increase in EdU labeling in transgenic zebrafish in both experiments, but this difference was not statistically significant in experiment 1. Only 4 zebrafish were examined for each group in experiment 1. Cell size was increased slightly in transgenic zebrafish versus non-transgenic zebrafish in experiment 1 but the difference was not statistically significant (N = 8 (Non-Tg) and N = 7 (Tg)). For cell size, quantification was done slightly differently in experiment 1; we measured 100 cells in slice 1 to calculate the average cell size for each larva.

To quantify Wnt reporter activity, we used *Tg(fabp10a*:*pt-β-cat)* ^*s986*^ *; Tg(7xTCF-Xla*.*Siam*:*GFP)* zebrafish or *Tg(fabp10a*:*pt-β-cat)* ^*s987*^ *; Tg(7xTCF-Xla*.*Siam*:*GFP)* zebrafish and *Tg(7xTCF-Xla*.*Siam*:*GFP)* control siblings. Larvae were fixed at 6 dpf, and nuclei were visualized by anti-PHH3 immunofluorescence staining or by incubating larvae for 30 minutes at room temperature in TO-PRO-3 stain (Life Technologies; 1:70 dilution) before rinsing and mounting. Larvae were blinded and imaged using the same parameters. Wnt reporter activity was quantified for each zebrafish by selecting a representative area of the liver using the anti-PHH3/TO-PRO-3/far red channel and quantifying the mean signal of the same area in the GFP/green channel using ImageJ. The Wnt reporter activity experiment was done at least twice for each drug dose with similar results; representative results from one experiment are shown.

To quantify *fabp10a* promoter activity, we fixed *Tg(fabp10a*:*pt-β-cat)* ^*s987*^ *; Tg(fabp10a*:*EGFP)* zebrafish and *Tg(fabp10a*:*EGFP)* control siblings at 6 dpf. Larvae were blinded and imaged using the same parameters. We quantified *fabp10a* promoter activity for each zebrafish by quantifying the mean signal in a representative area of the liver in the GFP/green channel using ImageJ. The *fabp10a* promoter activity experiment was done at least four times for each drug dose with similar results; representative results from one experiment are shown.

To quantify phospho-c-Jun staining in zebrafish larvae, we used *Tg(fabp10a*:*pt-β-cat)* ^*s987*^ zebrafish and sibling controls, treated with 5 µM SP600125, 5 μM EMD 420123 or 0.5% DMSO from 3–5 dpf. Larvae were fixed at 5 dpf and stained with phospho-c-Jun-specific antibody. Nuclei were visualized by incubating larvae for 30 minutes at room temperature in TO-PRO-3 stain (Life Technologies; 1:70 dilution) before rinsing and mounting. Larvae were imaged using the same parameters, the image files were blinded, and phospho-c-Jun staining for each larva was quantified by counting the total number of hepatocyte nuclei and the number of phospho-c-Jun-positive hepatocyte nuclei in the first slice of each image using ImageJ/FIJI. Phospho-jun staining of transgenic and non-transgenic larvae was done at least twice with qualitatively similar results, although only quantified in this manner once.

### Immunofluorescence and immunohistochemistry of adult liver sections

For immunofluorescence of adult zebrafish livers, representative samples of livers from 6–12-month-old *Tg(fabp10a*:*pt-β-cat)* ^*s986*^ zebrafish and control siblings were fixed in 4% PFA in PBS, incubated in 30% sucrose plus 0.02% sodium azide in PBS for at least 1 hour, and embedded in OCT compound. Cryosections were cut, air-dried, and stained with primary and secondary antibodies as described above, except Triton X-100 was omitted from rinsing and staining solutions and TO-PRO-3 stain was used at 1:500 dilution. Samples were imaged on a Zeiss LSM5 Pascal confocal microscope. Immunofluorescence experiments were done at least twice with qualitatively similar results.

For immunohistochemical analysis, we selected 3–6-month-old *Tg(fabp10a*:*pt-β-cat)* adult zebrafish livers that showed HCC on H&E-stained sections. As controls, we selected 3–6-month-old non-transgenic zebrafish that did not show substantial cytological or architectural abnormalities on H&E-stained sections. Immunohistochemistry was performed on formalin-fixed, paraffin-embedded tissue sections using phospho-c-Jun (Ser73) (D47G9) XP rabbit antibody (Cell Signaling Technology; Catalog Number 3270; 1:200 dilution) at the Gladstone Histology and Light Microscopy Core Facility following standard protocols including 0.3% H_2_O_2_ blocking, citrate steaming antigen retrieval, and counterstaining with Mayer’s hematoxylin. Slides were blinded, and the amount of nuclear staining was scored by a pathologist (K.J.E.) on a scale from 0–4+ based on the most intensely stained area of the slide: 0, no nuclear staining or rare scattered hepatocytes with nuclear staining; 1+, occasional scattered hepatocytes with nuclear staining; 2+, nuclear staining in 10–50% of hepatocytes; 3+, nuclear staining in greater than 50% of hepatocytes. For statistical analysis, 0 and 1+ staining patterns were considered phospho-c-Jun negative, and 2+ (moderate) and 3+ (strong) staining patterns were considered phospho-c-Jun positive.

### Quantitative real-time PCR of zebrafish livers

Representative portions of adult *Tg(fabp10a*:*pt-β-cat)* ^*s986*^ or *Tg(fabp10a*:*pt-β-cat)* ^*s987*^ zebrafish and non-transgenic control siblings age 1–12 months were frozen in TRIzol reagent (Life Technologies) and stored at -80°C until use. We extracted RNA, treated with RQ1 RNase free DNase (Promega, Madison, WI), purified using RNA Clean & Concentrator (Zymo Research, Irvine, CA), and measured concentration and purity using a NanoDrop 2000C Spectrophotometer (Thermo Scientific, West Palm Beach, FL). Up to 2 μg of RNA was reverse transcribed using a Superscript VILO cDNA synthesis kit (Life Technologies), and the resulting cDNA was diluted for quantitative real-time PCR (qRT-PCR) based on the starting RNA concentration. We designed primers for zebrafish *jun* (NM_199987) (5’-CCGACGTGGGACTTCTCAAA-3’; 5’-ATCCGTCACGTTCTTGGGAC-3’), zebrafish *ctnnb1* (NM_131059.2) (5’-GACAGGACGACCCAAGCTAC-3’; 5’-GCCGTCTACGGGGTAATCAG-3’), and *Xenopus laevis ctnnb1* (NM_001090576.1) (5’- GGCCACTCGAGCAATCCCCG-3’; 5’- AGCATGGCGTGAGGCTTCCT-3’) using Primer-BLAST (National Center for Biotechnology information); primers for β-actin (5’-GTGGTCTCGTGGATACCGCAA-3’; 5’-CTATGAGCTGCCTGACGGTCA-3’) have been described previously[79]. We performed qRT-PCR using Power SYBR Green Master Mix (Life Technologies/Applied Biosystems) and the 7900HT Real-Time PCR system (Life Technologies/Applied Biosystems)(*jun* analysis) or the Chromo4 Real-time PCR detection system with MJ Opticon Monitor Analysis Software (BioRad, Hercules, CA) (*ctnnb1* analysis). We determined the relative expression of *jun* or *ctnnb1* mRNA in each sample using the relative standard curve method[80]. Three technical replicates were performed for each sample; each sample represents a distinct animal. For zebrafish *jun* analysis, before statistical comparison of results, three samples were repeated and one sample was excluded because of pipetting errors, and only values for successful experiments are shown. For one zebrafish (T2), two samples were taken from different areas of the liver; relative *jun* mRNA expression was similar in the two samples, so results from only one representative sample are shown.

### Immunohistochemistry of human HCC samples

Human HCC specimens were selected from the pathology files of the University of California, San Francisco, and had been previously evaluated for nuclear β-catenin staining and glutamine synthetase expression by immunohistochemistry for clinical or research[81] purposes. Cases were defined as β-catenin active if the tumor showed diffuse strong glutamine synthetase staining (greater than 90% of tumor cells positive) or diffuse intermediate glutamine synthetase staining (50 to 90% of tumor cells positive). Cases were defined as β-catenin inactive if the tumor showed glutamine synthetase staining in less than 50% of tumor cells. Immunohistochemistry for phospho-c-Jun was performed on formalin-fixed, paraffin-embedded tissue sections using phospho-c-Jun (Ser73) (D47G9) XP rabbit antibody (Cell Signaling Technology; Catalog Number 3270; 1:200 dilution) at the Gladstone Histology and Light Microscopy Core Facility as described above. Slides were scored in a blinded fashion by K.J.E. and confirmed by a second pathologist (S.K.); cases were considered phospho-c-Jun positive if definitive nuclear staining was present in any tumor cells.

### Analysis of *JUN* expression in human HCC samples

We used Microsoft Excel to sort genome-wide expression profiles of 96 human HCC samples[39] based on *GLUL* expression levels. We defined “*GLUL* high” tumors as the 32 tumors with the highest *GLUL* expression, and “*GLUL* low” tumors as the 32 tumors with the lowest *GLUL* expression. We normalized *JUN* expression levels for each of tumor sample by subtracting the average *JUN* expression level of all 96 tumors.

### Cell culture, cell viability assays, and western blotting analysis

Human liver cancer cell lines were obtained from the American Type Culture Collection (ATCC, Manassas, VA)(Snu-398, Snu-423, Snu-449), Riken Bioresource Center Cell Bank (Osaka, Japan) (Huh-6), or UCSF Cell Culture Facility (Huh-7 and HepG2) and cultured in DMEM plus 10% fetal bovine serum. Immortalized human hepatocytes (CRL-4020) were obtained from the ATCC and cultured in DMEM:F12 plus 10% fetal bovine serum, 50 μg/mL G-418, 0.1 μL/mL dexamethasone (Lonza, Allendale, NJ), and 0.11 μL/mL insulin (Lonza) in collagen-coated flasks. Adherent cells were treated for 3 days with DMSO (control), CC401 (Santa Cruz Biotechnology, Dallas, TX), or amitriptyline (Sigma-Aldrich) dissolved in DMSO. To determine the dose at which CC401 or amitriptyline inhibited growth by 50% (GI50), for each experiment cells were treated with 4–8 serial dilutions of drug with 6 replicates per concentration. CellTiter-Glo (Promega) Luminescent Cell Viability Assay was performed in 96-well plates, using a Safire2 plate reader (TECAN) and Magellan software. GI50 for each experiment was determined using GraphPad Software, and at least three experiments were performed for each drug for each cell line. For Western blot analysis, cultured cells were washed with PBS and collected into radioimmunoprecipitation assay (RIPA) buffer containing protease and phosphatase inhibitors as described [82]. Protein concentrations were determined using DC Protein Assay (Bio-Rad Laboratories, Hercules, CA) with bovine serum albumin standards; equal loading was confirmed by β-actin and Ponceau S staining of each membrane.

### Mouse hydrodynamic transfection and drug treatment

Hydrodynamic transfection (HDT) was performed as described previously[42]. Female FVB/N mice, obtained from Taconic Biosciences (Hudson, NY) or Jackson Laboratory (Bar Harbor, ME), were injected at 6 to 8 weeks of age with 100 μL per gram of 0.9% normal saline containing plasmids encoding Sleeping Beauty transpose (800 ng/mL), human *MET* (10 μg/mL), and *ΔN90-CTNNB1* (10 μg/mL)[42]. One month post-HDT, at 10 to 12 weeks of age, the animals were randomized into control or experimental groups and their drinking water was replaced with sterile water containing 200 mg/L amitriptyline plus 0.5–2% saccharine (experimental) or sterile water containing 0.5–2% saccharine alone (control). Mice were sacrificed by regulated administration of carbon dioxide followed by dissection of a major organ (liver) approximately 13 weeks post-HDT, when palpable tumors were present in control animals, except for two control mice and one amitriptyline-treated mouse, which were found dead at 13 weeks post-HDT. Within each experiment, the same concentration of saccharine was used for all mice and mice were sacrificed at the same time point. Four independent experiments were pooled. Three control mice and five amitriptyline-treated mice were excluded from analysis because of very low tumor burden, indicating unsuccessful HDT. One amitriptyline-treated mouse was excluded from analysis because it was found dead.

As non-HDT control animals, 11-week-old female FVM/N mice were obtained from Taconic Biosciences and given sterile water containing 200 mg/L amitriptyline plus 0.5% saccharine or sterile water containing 0.5% saccharine alone. After 12 weeks, mice were sacrificed by regulated administration of carbon dioxide followed by dissection of a major organ (liver).

### Immunohistochemistry of murine samples

Mouse livers were fixed in 4% paraformaldehyde in PBS. Paraffin-embedded blocks and hematoxylin-and-eosin-stained slides were prepared at the Gladstone Histology and Light Microscopy Core Facility. All HDT animals included in the liver and body weight analyses were analyzed by immunohistochemistry, except for three animals (two control mice and one amitriptyline-treated mouse), which were found dead at 13 weeks post-HDT. TUNEL assay was performed using the ApopTag Peroxidase In Situ Apoptosis Detection Kit (EMD Millipore/Calbiochem) following the manufacturer's protocol. Immunohistochemistry for Ki-67 was performed using pre-diluted Ki-67 antibody (Thermo Scientific; Catalog Number RM-9106-R7) following deparaffinization, rehydration, quenching of endogenous peroxidase activity by hydrogen peroxide incubation, and blocking in 1.5% normal goat serum. Goat anti-rabbit IgG-Biotin secondary antibody was used (Santa Cruz; Catalog Number sc-2040). Samples were detected with VECTASTAIN Elite ABC Reagent (Vector Labs) and Vector DAB substrate kit (Vector Labs) and counterstained with hematoxylin. To quantify proliferation and cell death, slides were blinded and a pathologist (K.J.E.) counted the number of Ki-67- or TUNEL-positive cells in 10–30 high-power fields for each mouse. Photographs of mouse liver sections were taken using an Olympus BX53 microscope with UPlanSAPpo objectives, Olympus DP72 camera, and Olympus cellSens Standard software.

### Statistical methods

Student’s two-tailed t-tests were performed using Microsoft Excel. Fisher’s exact tests, Mann-Whitney tests, and 2-way ANOVA were performed using GraphPad Prism 6 Software. Survival curves were generated and analyzed using MedCalc statistical software and GraphPad Software. Comparisons were considered statistically significant if p-values were less than 0.05. Standard error of the mean is reported for each group of data. In general, sample size and variance were similar between all groups being statistically compared.

### Ethics statement

Animal studies were carried out following guidelines and standard procedures set forth by the University of California at San Francisco Institutional Animal Care and Use Committee (UCSF IACUC). The UCSF IACUC approved all protocols involving live vertebrate animals (AN106527, AN090555). Zebrafish were euthanized by tricaine or rapid chilling, following UCSF IACUC guidelines. Mice were euthanized by carbon dioxide inhalation/administration followed by cervical dislocation and/or dissection of a major organ (liver), following UCSF IACUC guidelines. Human studies were approved by the institutional review board of UCSF, the Human Research Protection Program Committee on Human Research (IRB #10–04369). The requirement for informed consent was waived because the research involved at most minimal risk to the subjects, the waiver would not adversely affect therights and/or welfare of the subjects, the research could not practically be carried out without the waiver, and the subjects would be provided with additional pertinent information after participation, if appropriate.

## Supporting Information

S1 Fig *Tg(fabp10a*:*pt-β-cat)* zebrafish express activated β-catenin. (**A**) Plasmid encoding hepatocyte-specific pt-β-catenin and a fluorescent eye marker was injected into embryos to generate *Tg(fabp10a*:*pt-β-cat*, *cryaa*:*Venus)* zebrafish. (**B-C**) Normalized transgenic *Xenopus* (**B**) and endogenous zebrafish (**C**) *ctnnb1* mRNA expression in control and transgenic adult zebrafish livers. Three technical replicates were performed for each sample. Groups were compared using Mann-Whitney test. N values are shown above the x-axis. (**D**) Immunofluorescence of adult control sibling zebrafish liver cryosections (top panels) show membrane localization of β-catenin, whereas transgenic zebrafish livers show patchy cytoplasmic β-catenin staining and scattered β-catenin-positive nuclei (arrows). (**E**) Similarly, whole-mounted 5-day-old control sibling larvae (top row) show membrane localization of β-catenin while transgenic zebrafish (bottom row) show patchy strong cytoplasmic and nuclear β-catenin staining. Immunofluorescent staining for β-catenin was performed in *Tg(fabp10a*:*EGFP)* larvae, which have green hepatocytes; merged images show β-catenin is expressed in hepatocytes. (**F**) Six-day-old control sibling larvae (left) show essentially no *7xTCF-Xla*.*Siam*:GFP (Wnt reporter) expression in hepatocytes, while transgenic larvae (right) show scattered hepatocytes with moderate to strong GFP positivity. (**G**) Fluorescence intensity ± standard error of the mean (SEM) was quantified using ImageJ, and samples were compared using the Student’s *t*-test (***, p<0.001). Scale bars, 20 μm. N values are shown above the x-axis.
(TIFF)

S2 Fig Zebrafish livers with activated β-catenin share histologic features with human hepatocellular carcinoma (HCC). **(A)** Adult zebrafish livers with activated β-catenin (left) and human HCC (right) show architectural disruption including enlarged interhepatic spaces resembling spongiosis hepatis (zebrafish) or peliosis hepatis (human) (asterisks). **(B-D)** Similarly, zebrafish (left) and human (right) samples show cytological abnormalities including nuclear enlargement and nuclear contour irregularities (arrows, **B**), prominent nucleoli (arrows, **C**), and mitotic figures (arrows, **D**). Hematoxylin and eosin (H&E) stained sections; scale bars, 20 μm.
(TIFF)

S3 Fig Effect of activated β-catenin on zebrafish body mass. Graph showing mean body mass ± SEM. Asterisks indicate p-values for ANOVA comparing transgenic zebrafish to control siblings at the same time point: **, p<0.01 (4 months post fertilization). Other comparisons were not statistically significant. N values are shown above the x-axis.
(TIFF)

S4 Fig *Tg(fabp10a*:*pt-β-cat)* zebrafish show DNA aneuploidy. **(A-D)** Flow cytometric plots showing DNA content, quantified by propidium iodide staining, for four representative non-transgenic control zebrafish livers. All plots show a dominant peak at 2N (arrows) with a smaller peak at 4N (arrowheads). In (**C-D**), samples were spiked with sperm to provide 1N peak as reference (shaded in red). (**E-H**) Flow cytometric plots showing DNA content, quantified by propidium iodide staining, for four representative *Tg(fabp10a*:*pt-β-cat)* zebrafish livers showing evidence of DNA aneuploidy. (**E**) Large peak between 2N and 4N (arrowhead). (**F**) Broadened, multiple peaks near 4N and >4N (bracket). (**G**) Large peak between 2N and 4N (arrowhead). (**H**) Broadened double peak near 4N (bracket). In (**G-H**), samples were spiked with sperm to provide 1N peak as reference (shaded in red).
(TIFF)

S5 Fig Ingenuity pathways analysis (IPA) of differentially expressed genes in *Tg(fabp10a*:*pt-β-cat)* zebrafish compared to non-transgenic control siblings. Microarray analysis was performed on 4-month-old transgenic zebrafish and control siblings, and average fold-changes of probes with significant signals above background were inputted into IPA with a fold-change cut-off of 2.0. Figure shows summary provided by Ingenuity Systems.
(TIFF)

S6 Fig Small molecule screen for compounds that suppress larval liver enlargement caused by activated β-catenin. Transgenic zebrafish expressing activated β-catenin (*Tg(fabp10a*:*pt-β-cat*, *cryaa*:*Venus*)) were crossed to zebrafish expressing liver-specific GFP (*Tg(fabp10a*:*EGFP*). At 3 days old, larvae with green livers (*fabp10a*:EGFP+) were selected for drug treatment. Zebrafish with activated β-catenin, identified by their fluorescent eyes, were treated in parallel to control zebrafish without activated β-catenin. Three zebrafish were placed in each well, and 4 wells were tested for each experimental compound and vehicle (DMSO) control. Liver size was scored qualitatively 3 days post treatment (dpt). Drugs that decreased average liver size of *Tg(fabp10a*:*pt-β-cat); Tg(fabp10a*:*EGFP)* zebrafish in both wells compared to DMSO controls without causing toxicity/death in any wells were considered potential hit compounds.
(TIFF)

S7 Fig SP600125 and amitriptyline do not significantly affect *fabp10a* promoter activity or Wnt reporter activity. (**A**) Representative photographs of *fabp10a*:EGFP expression (the same *fabp10a* promoter element used to drive *pt-β-cat* expression) in control (top row) and *Tg(fabp10a*:*pt-β-cat)* (bottom row) zebrafish livers at 5 dpf. (**B**) Fluorescence intensity ± standard error of the mean (SEM) was quantified using ImageJ, and samples were compared using 2-way ANOVA; p>0.05 for each group compared to every other group. N values are shown above the x-axis. (**C**) Representative photographs of *7xTCF-Xla*.*Siam*:GFP (Wnt reporter) expression in control (top row) and *Tg(fabp10a*:*pt-β-cat)* (bottom row) zebrafish livers at 6 dpf. (**D**) Fluorescence intensity ± standard error of the mean (SEM) was quantified using ImageJ, and samples were compared using 2-way ANOVA (p>0.05 for all drug treatments compared to DMSO control of same genotype.) Scale bars, 20 μm. Six zebrafish were analyzed for each group.
(TIFF)

S8 Fig Effect of amitriptyline on human liver cancer cell lines. Graph showing dose of amitriptyline at which cell viability of immortalized human hepatocytes (HepTert) and human liver cancer cell lines was decreased by 50% (GI50), ± SEM. The difference between β-catenin wild-type (WT) and β-catenin mutant cell lines was not statistically significant (p>0.05, unpaired t-test). However, 5 out of 6 human liver cancer cell lines had a significantly lower GI50 than human non-tumor liver (HepTert) cells. Asterisks indicate p-values for one-way ANOVA comparing each human liver cancer cell line to HepTert cells: *, p<0.05; **, p<0.01. Number of replicates for each cell line is shown above the x-axis.
(TIFF)

S9 Fig Histology of mice hydrodynamically transfected with activated β-catenin and Met. (**A**) Representative images of livers from control, non-hydrodynamically transfected (non-HDT) mice. Mice were treated with saccharine alone (vehicle only, top row) or amitriptyline plus saccharine (bottom row). Sections show an orderly arrangement of hepatocytes, without cytological atypia. (**B**) Representative images of mice hydrodynamically transfected with activated β-catenin and Met (Met/β-cat HDT). Mice were treated with saccharine alone (vehicle only, top row) or amitriptyline plus saccharine (bottom row). Sections show innumerable coalescing tumors characterized by disorganized plate architecture. Scale bars, 200 μm.
(TIFF)

S10 Fig Effect of amitriptyline on liver mass and body mass. (**A**) Graph showing mean liver mass ± SEM for non-HDT and Met/β-cat HDT mice treated with saccharine alone (vehicle) or amitriptyline plus saccharine (+Ami). P values calculated with Mann-Whitney test. (**B**) Graph showing mean body mass ± SEM for non-HDT and Met/β-cat HDT treated with saccharine alone (vehicle) or amitriptyline plus saccharine (+Ami). P values calculated with Mann-Whitney test. The number of mice in each group is shown above the x-axis.
(TIFF)

S11 Fig Additional representative hematoxylin-and-eosin-stained (H&E), Ki-67-labeled, and TUNEL-labeled images from mouse liver tumors induced by hydrodynamic transfection of activated β-catenin and Met. Mice were treated with saccharine alone **(A)** or amitriptyline plus saccharine **(B)**. Ki-67 and TUNEL staining were performed using 3, 3'-diaminobenzidine (DAB) substrate, so positive-staining cells are brown, and hematoxylin counterstain, to highlight nuclei and other basophilic structures in blue. Scale bars, 20 μm. Quantification of this experiment is shown in Fig 6G and6H.
(TIFF)

S1 Dataset Clustering and pathway enrichment analyses of *Tg(fabp10a*:*pt-β-cat)* zebrafish and human HCC. (**A**) Merged_human_zebrafish_genes. These 283 orthologous gene pairs were significantly dys-regulated in human HCC samples when compared to adjacent non-tumor liver samples and were used for clustering analysis. (**B**) GOI_summary. These 485 human and zebrafish orthologs (genes of interest, GOI) were significantly dys-regulated in the same direction in both human HCC samples and *Tg(fabp10a*:*pt-β-cat)* animals when compared to their non-tumor counterparts and were used for pathway enrichment analyses. (**C**) GO_ora_cond_analysis. Results of pathway enrichment analysis of GOI using Gene Ontology (GO) terms annotations. (**D**) KEGG_ora_analysis. Results of pathway enrichment analysis of GOI using Kyoto Encyclopedia of Genes and Genomes (KEGG) database. (**E**) Reactome_ora_analysis. Results of pathway enrichment analysis of GOI using Reactome database.
(XLSX)

S1 Table Transgenic zebrafish strains used in this study. (DOCX)

S2 Table Comparison of diagnostic features of human hepatocellular carcinoma (HCC) and criteria used to diagnose HCC in zebrafish. (DOCX)

S3 Table Body mass, liver mass, and liver-to-body mass ratios of *Tg(fabp10a*:*pt-β-cat)* zebrafish and sibling controls. (DOCX)

S4 Table Key pathways in *Tg(fabp10a*:*pt-β-cat)* zebrafish and human HCC. (DOCX)

S5 Table Hit compounds that suppressed larval liver enlargement caused by activated β-catenin in zebrafish larvae. (DOCX)
