# Supplementary figures and images for: Identification of Chemical Inhibitors of β-Catenin-Driven Liver Tumorigenesis in Zebrafish
Source: PLoS Genet. 2015 Jul 2;11(7):e1005305. doi: 10.1371/journal.pgen.1005305 (PMC4489858; doi:10.1371/journal.pgen.1005305)

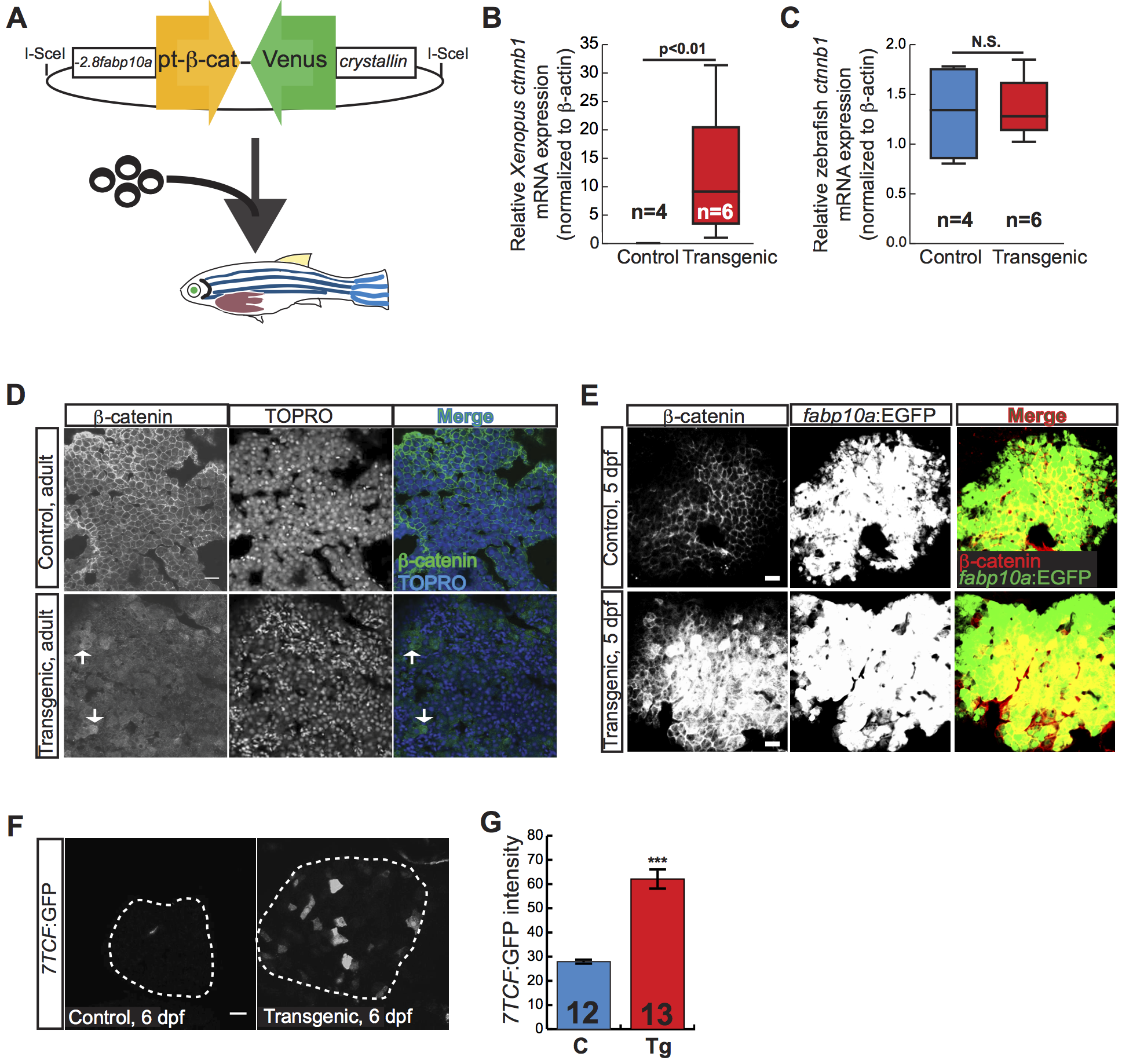

Supplement: S1 Fig — (A) Plasmid encoding hepatocyte-specific pt-β-catenin and a fluorescent eye marker was injected into embryos to generate Tg(fabp10a:pt-β-cat, cryaa:Venus) zebrafish. (B-C) Normalized transgenic Xenopus (B) and endogenous zebrafish (C) ctnnb1 mRNA expression in control and transgenic adult zebrafish livers. Three technical replicates were performed for each sample. Groups were compared using Mann-Whitney test. N values are shown above the x-axis. (D) Immunofluorescence of adult control sibling zebrafish liver cryosections (top panels) show membrane localization of β-catenin, whereas transgenic zebrafish livers show patchy cytoplasmic β-catenin staining and scattered β-catenin-positive nuclei (arrows). (E) Similarly, whole-mounted 5-day-old control sibling larvae (top row) show membrane localization of β-catenin while transgenic zebrafish (bottom row) show patchy strong cytoplasmic and nuclear β-catenin staining. Immunofluorescent staining for β-catenin was performed in Tg(fabp10a:EGFP) larvae, which have green hepatocytes; merged images show β-catenin is expressed in hepatocytes. (F) Six-day-old control sibling larvae (left) show essentially no 7xTCF-Xla.Siam:GFP (Wnt reporter) expression in hepatocytes, while transgenic larvae (right) show scattered hepatocytes with moderate to strong GFP positivity. (G) Fluorescence intensity ± standard error of the mean (SEM) was quantified using ImageJ, and samples were compared using the Student’s t-test (***, p<0.001). Scale bars, 20 μm. N values are shown above the x-axis. (TIFF) [file pgen.1005305.s001.tiff]

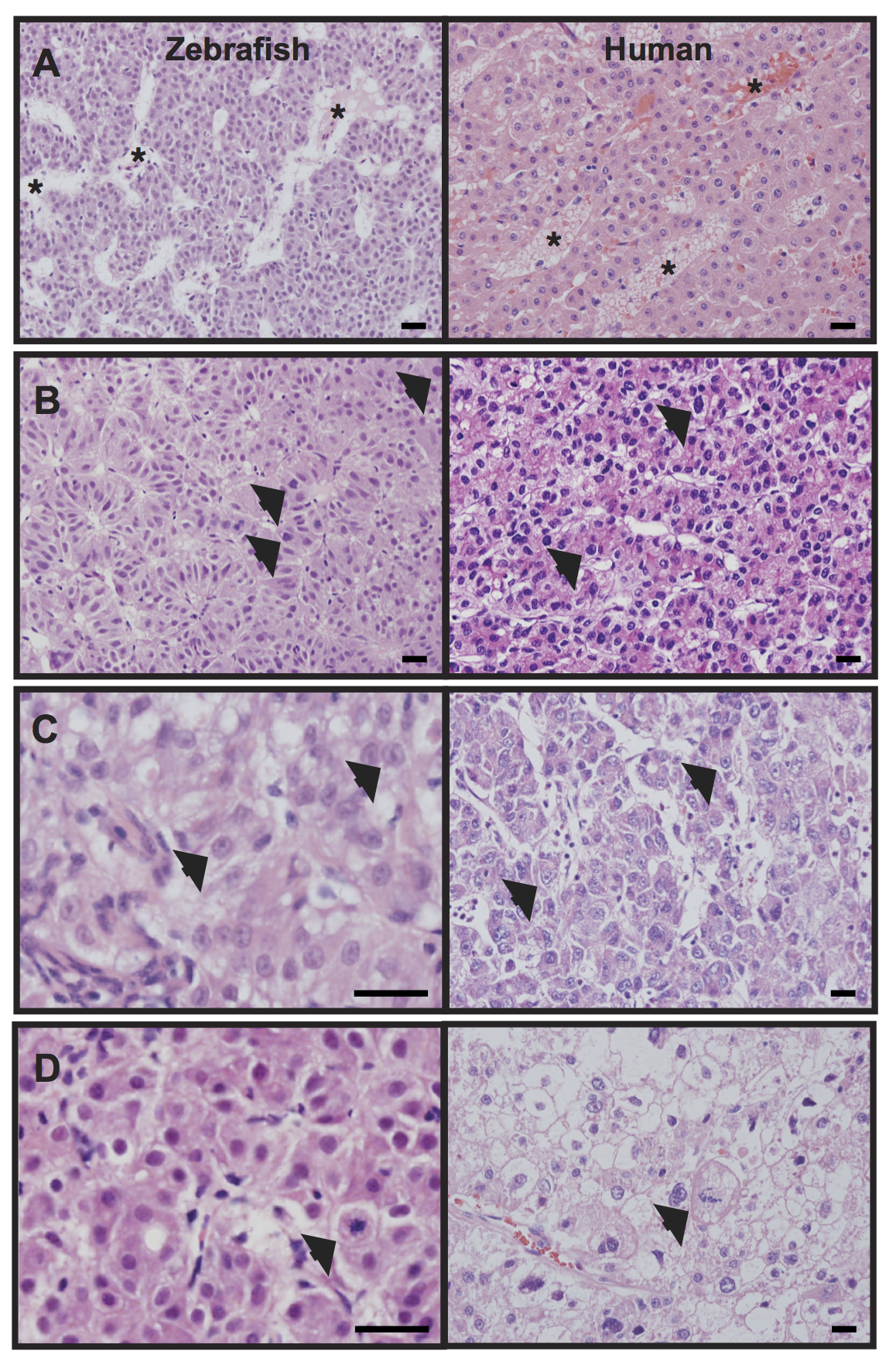

Supplement: S2 Fig — (A) Adult zebrafish livers with activated β-catenin (left) and human HCC (right) show architectural disruption including enlarged interhepatic spaces resembling spongiosis hepatis (zebrafish) or peliosis hepatis (human) (asterisks). (B-D) Similarly, zebrafish (left) and human (right) samples show cytological abnormalities including nuclear enlargement and nuclear contour irregularities (arrows, B), prominent nucleoli (arrows, C), and mitotic figures (arrows, D). Hematoxylin and eosin (H&E) stained sections; scale bars, 20 μm. (TIFF) [file pgen.1005305.s002.tiff]

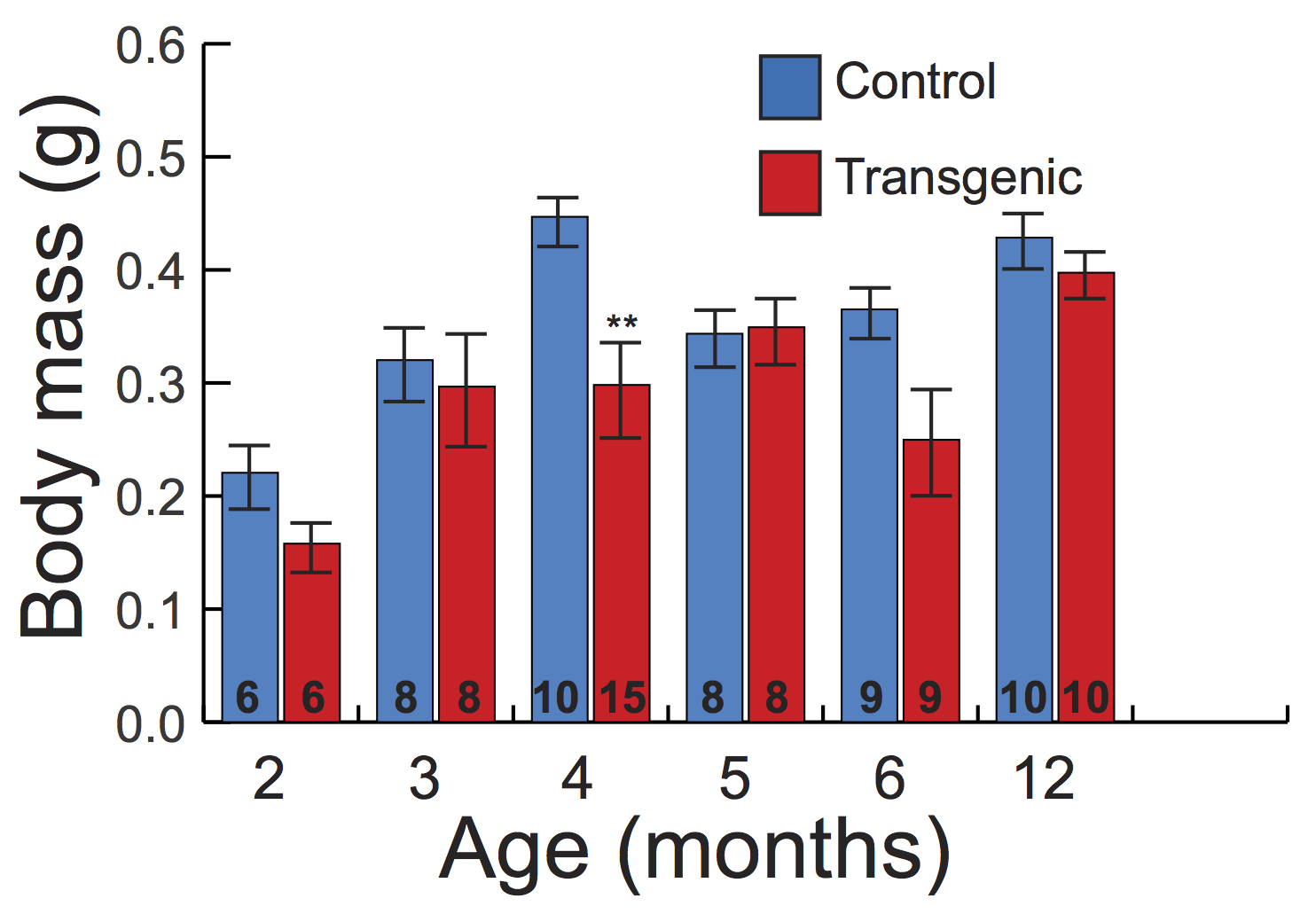

Supplement: S3 Fig — Graph showing mean body mass ± SEM. Asterisks indicate p-values for ANOVA comparing transgenic zebrafish to control siblings at the same time point: **, p<0.01 (4 months post fertilization). Other comparisons were not statistically significant. N values are shown above the x-axis. (TIFF) [file pgen.1005305.s003.tiff]

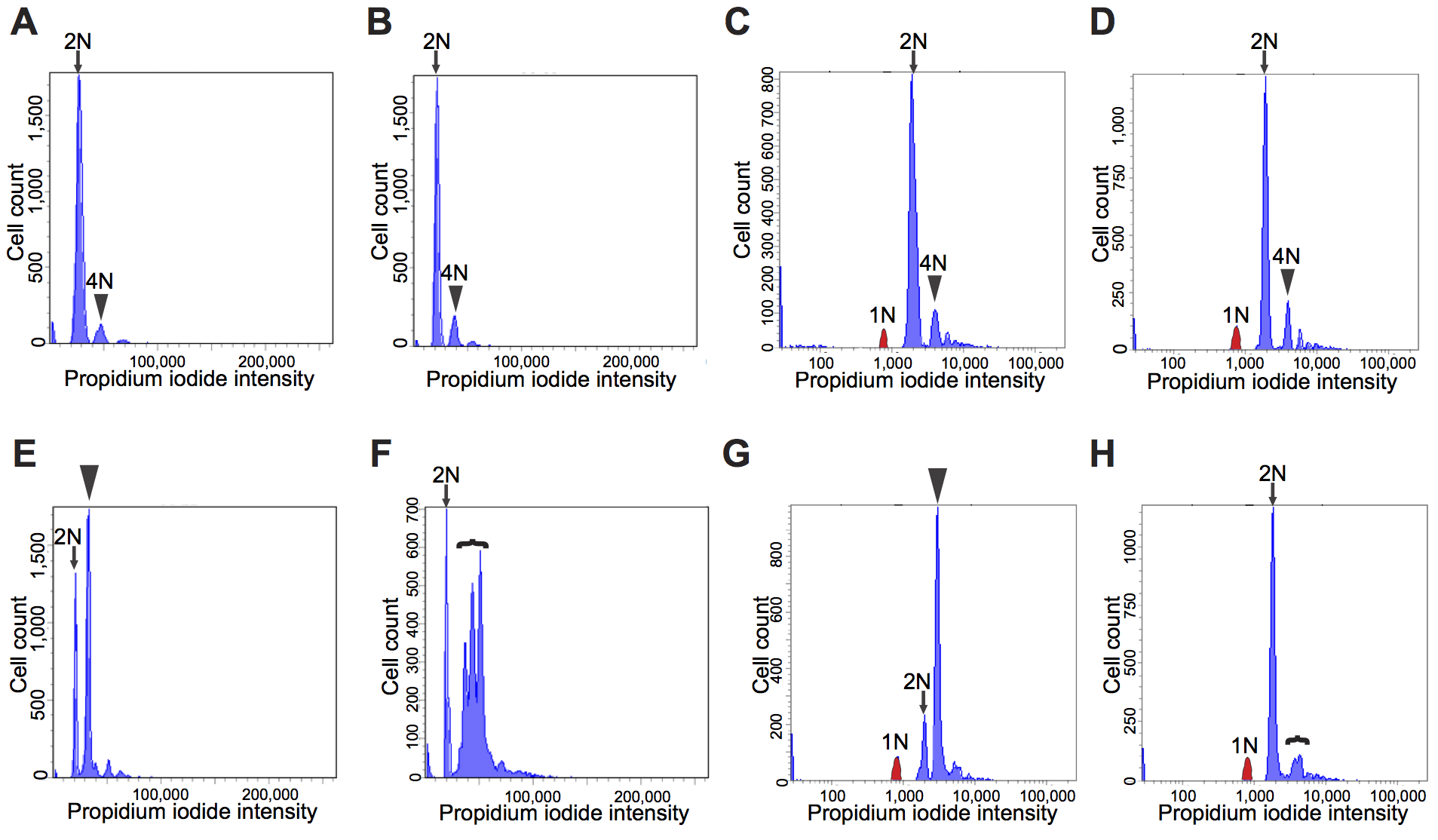

Supplement: S4 Fig — (A-D) Flow cytometric plots showing DNA content, quantified by propidium iodide staining, for four representative non-transgenic control zebrafish livers. All plots show a dominant peak at 2N (arrows) with a smaller peak at 4N (arrowheads). In (C-D), samples were spiked with sperm to provide 1N peak as reference (shaded in red). (E-H) Flow cytometric plots showing DNA content, quantified by propidium iodide staining, for four representative Tg(fabp10a:pt-β-cat) zebrafish livers showing evidence of DNA aneuploidy. (E) Large peak between 2N and 4N (arrowhead). (F) Broadened, multiple peaks near 4N and >4N (bracket). (G) Large peak between 2N and 4N (arrowhead). (H) Broadened double peak near 4N (bracket). In (G-H), samples were spiked with sperm to provide 1N peak as reference (shaded in red). (TIFF) [file pgen.1005305.s004.tiff]

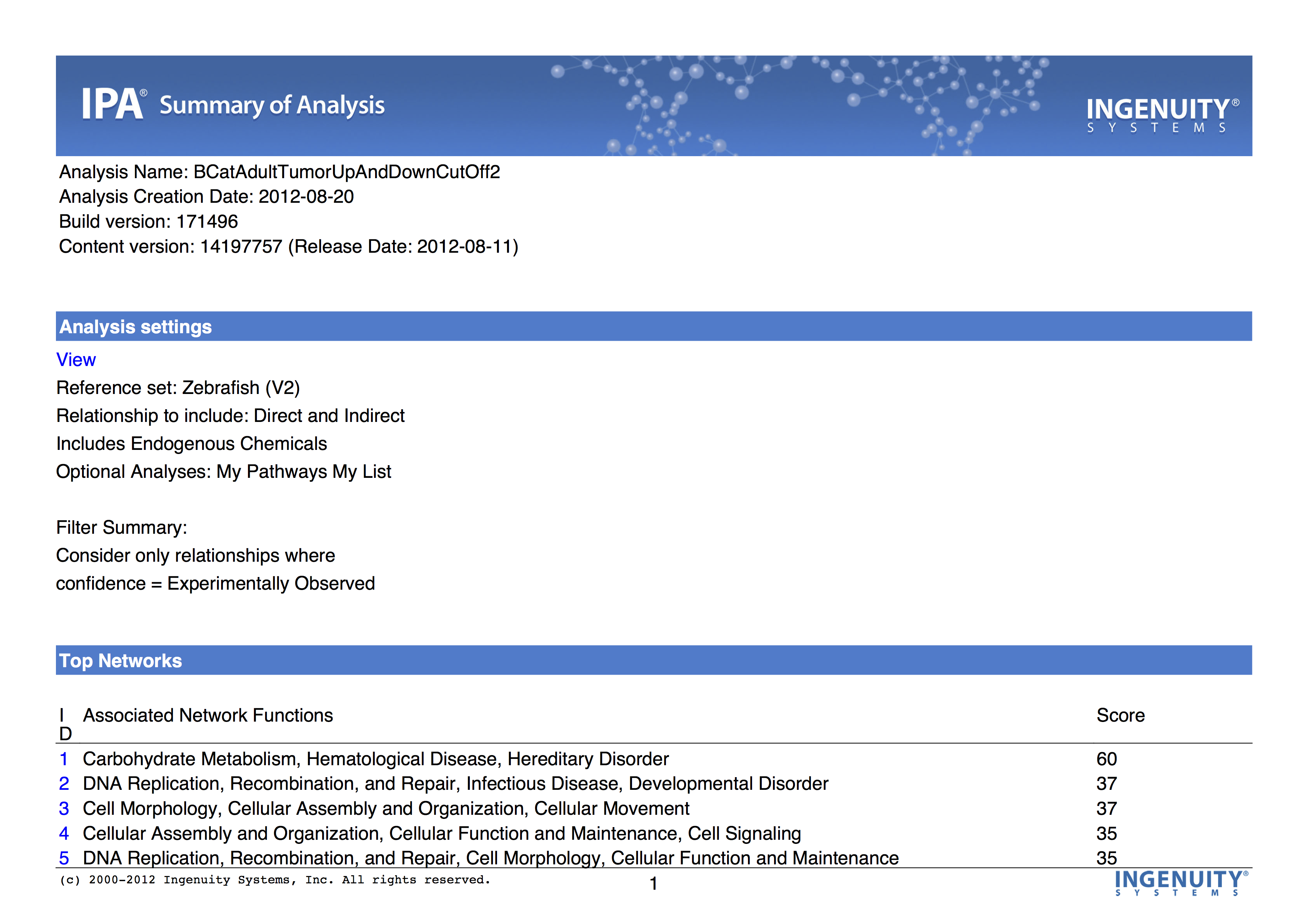

Supplement: S5 Fig — Microarray analysis was performed on 4-month-old transgenic zebrafish and control siblings, and average fold-changes of probes with significant signals above background were inputted into IPA with a fold-change cut-off of 2.0. Figure shows summary provided by Ingenuity Systems. (TIFF) [file pgen.1005305.s005.tiff]

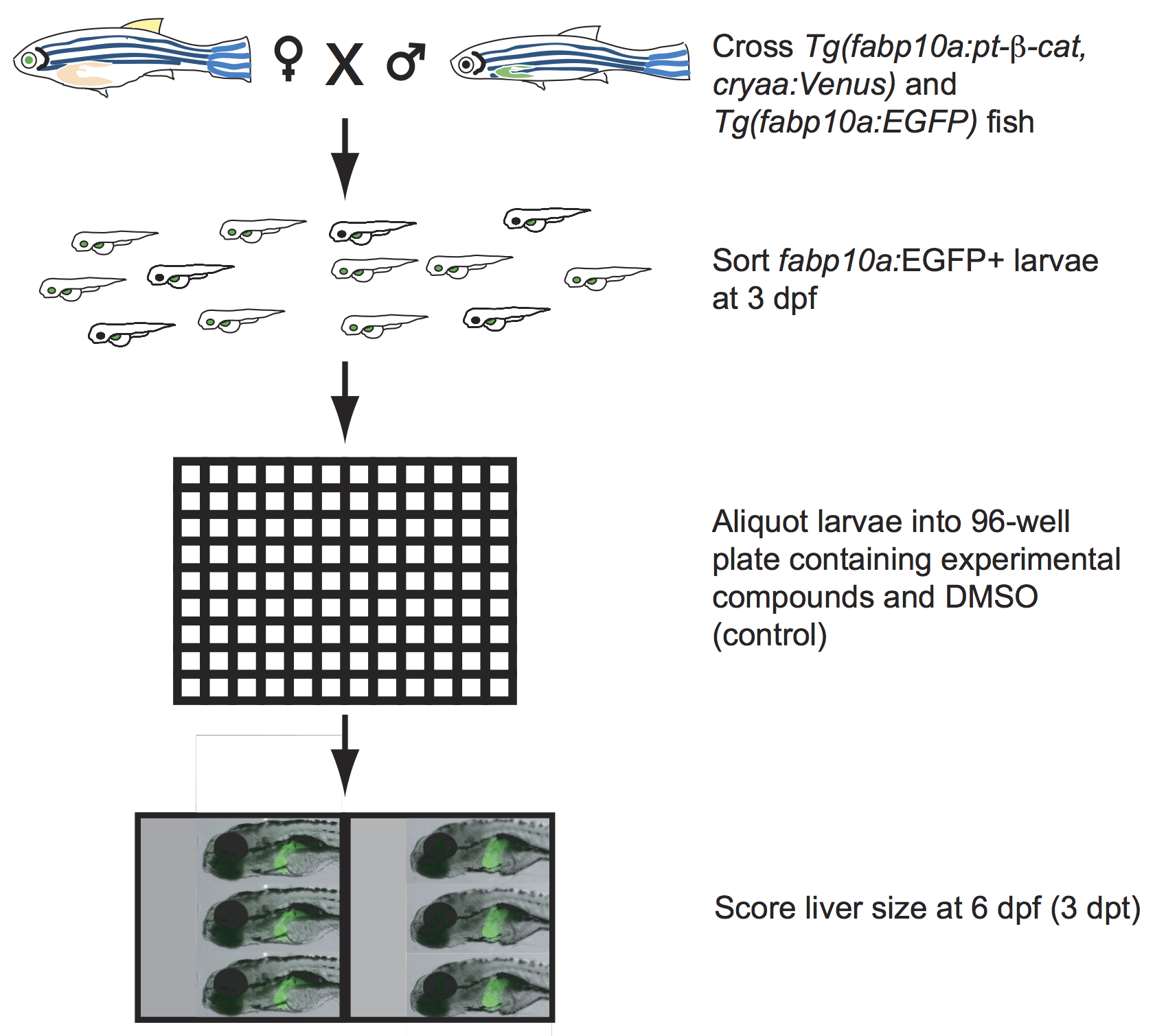

Supplement: S6 Fig — Transgenic zebrafish expressing activated β-catenin (Tg(fabp10a:pt-β-cat, cryaa:Venus)) were crossed to zebrafish expressing liver-specific GFP (Tg(fabp10a:EGFP). At 3 days old, larvae with green livers (fabp10a:EGFP+) were selected for drug treatment. Zebrafish with activated β-catenin, identified by their fluorescent eyes, were treated in parallel to control zebrafish without activated β-catenin. Three zebrafish were placed in each well, and 4 wells were tested for each experimental compound and vehicle (DMSO) control. Liver size was scored qualitatively 3 days post treatment (dpt). Drugs that decreased average liver size of Tg(fabp10a:pt-β-cat); Tg(fabp10a:EGFP) zebrafish in both wells compared to DMSO controls without causing toxicity/death in any wells were considered potential hit compounds. (TIFF) [file pgen.1005305.s006.tiff]

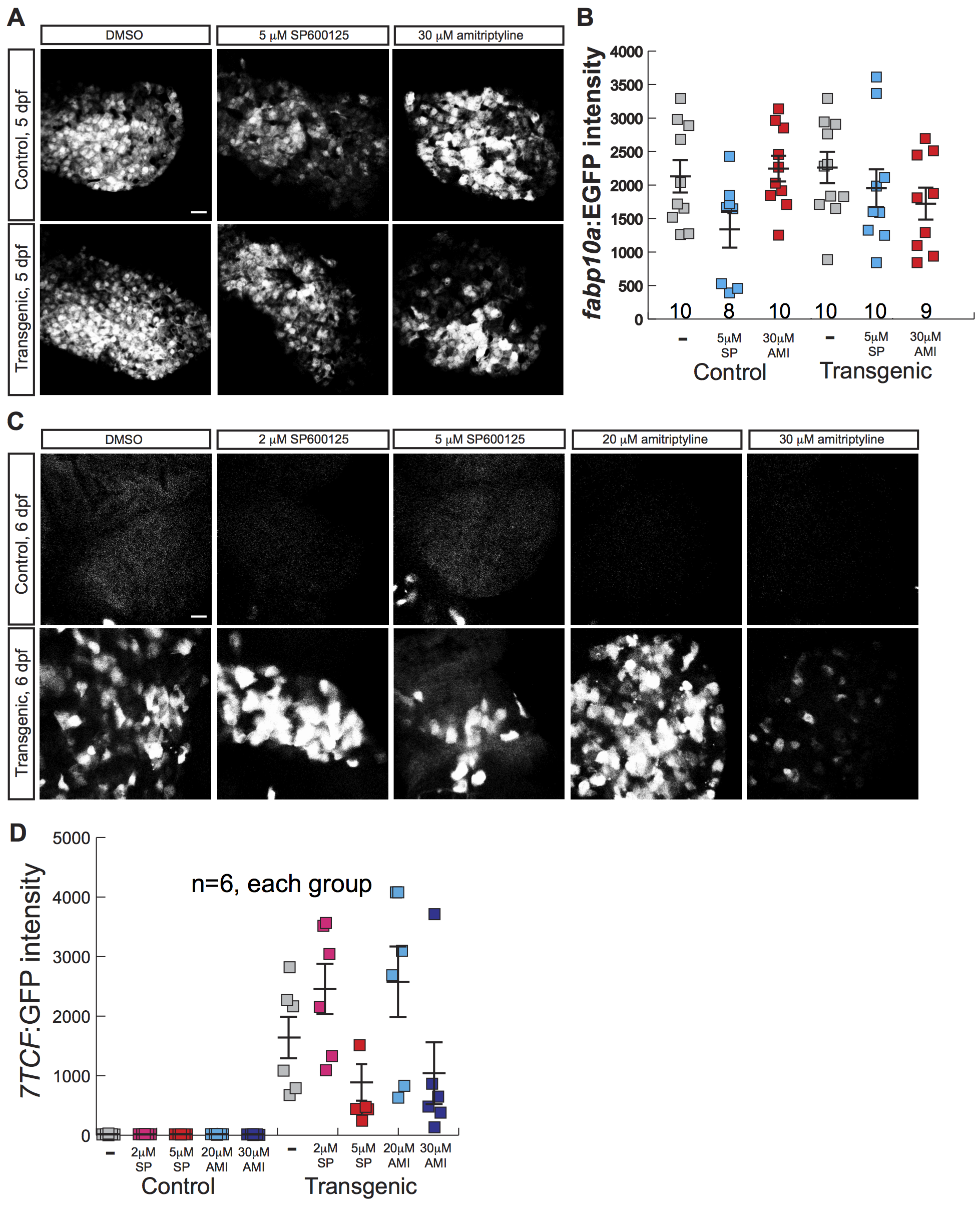

Supplement: S7 Fig — (A) Representative photographs of fabp10a:EGFP expression (the same fabp10a promoter element used to drive pt-β-cat expression) in control (top row) and Tg(fabp10a:pt-β-cat) (bottom row) zebrafish livers at 5 dpf. (B) Fluorescence intensity ± standard error of the mean (SEM) was quantified using ImageJ, and samples were compared using 2-way ANOVA; p>0.05 for each group compared to every other group. N values are shown above the x-axis. (C) Representative photographs of 7xTCF-Xla.Siam:GFP (Wnt reporter) expression in control (top row) and Tg(fabp10a:pt-β-cat) (bottom row) zebrafish livers at 6 dpf. (D) Fluorescence intensity ± standard error of the mean (SEM) was quantified using ImageJ, and samples were compared using 2-way ANOVA (p>0.05 for all drug treatments compared to DMSO control of same genotype.) Scale bars, 20 μm. Six zebrafish were analyzed for each group. (TIFF) [file pgen.1005305.s007.tiff]

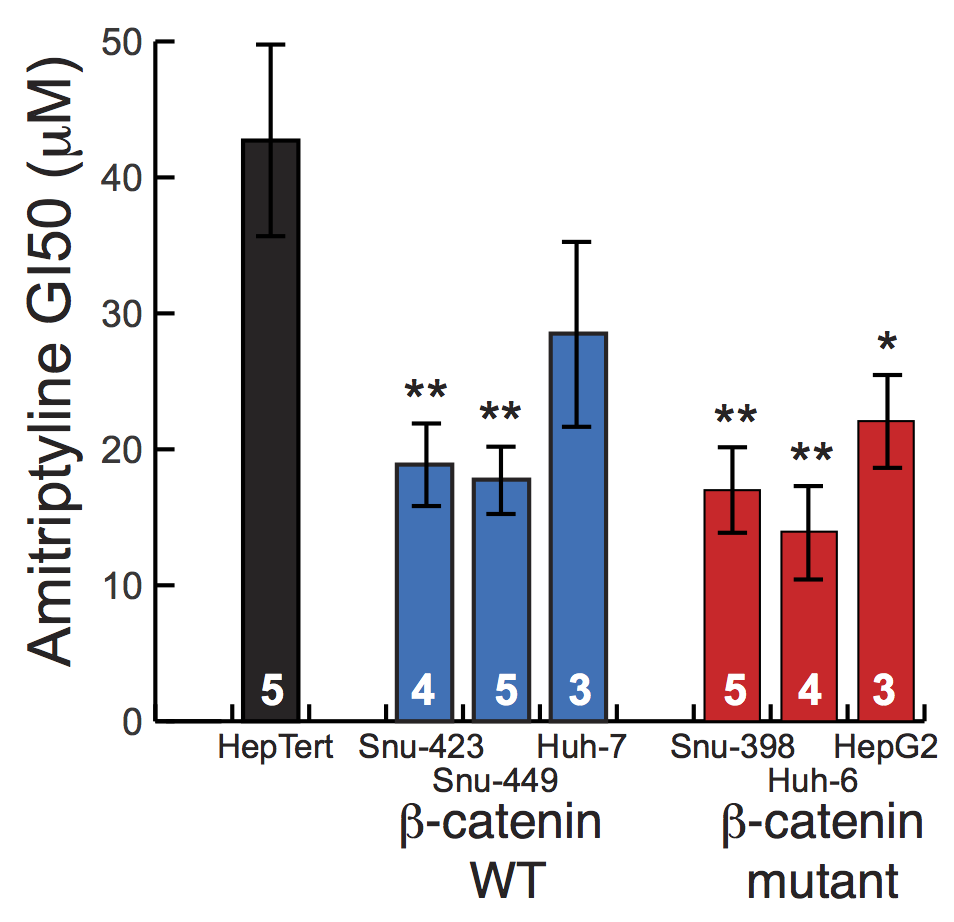

Supplement: S8 Fig — Graph showing dose of amitriptyline at which cell viability of immortalized human hepatocytes (HepTert) and human liver cancer cell lines was decreased by 50% (GI50), ± SEM. The difference between β-catenin wild-type (WT) and β-catenin mutant cell lines was not statistically significant (p>0.05, unpaired t-test). However, 5 out of 6 human liver cancer cell lines had a significantly lower GI50 than human non-tumor liver (HepTert) cells. Asterisks indicate p-values for one-way ANOVA comparing each human liver cancer cell line to HepTert cells: *, p<0.05; **, p<0.01. Number of replicates for each cell line is shown above the x-axis. (TIFF) [file pgen.1005305.s008.tiff]

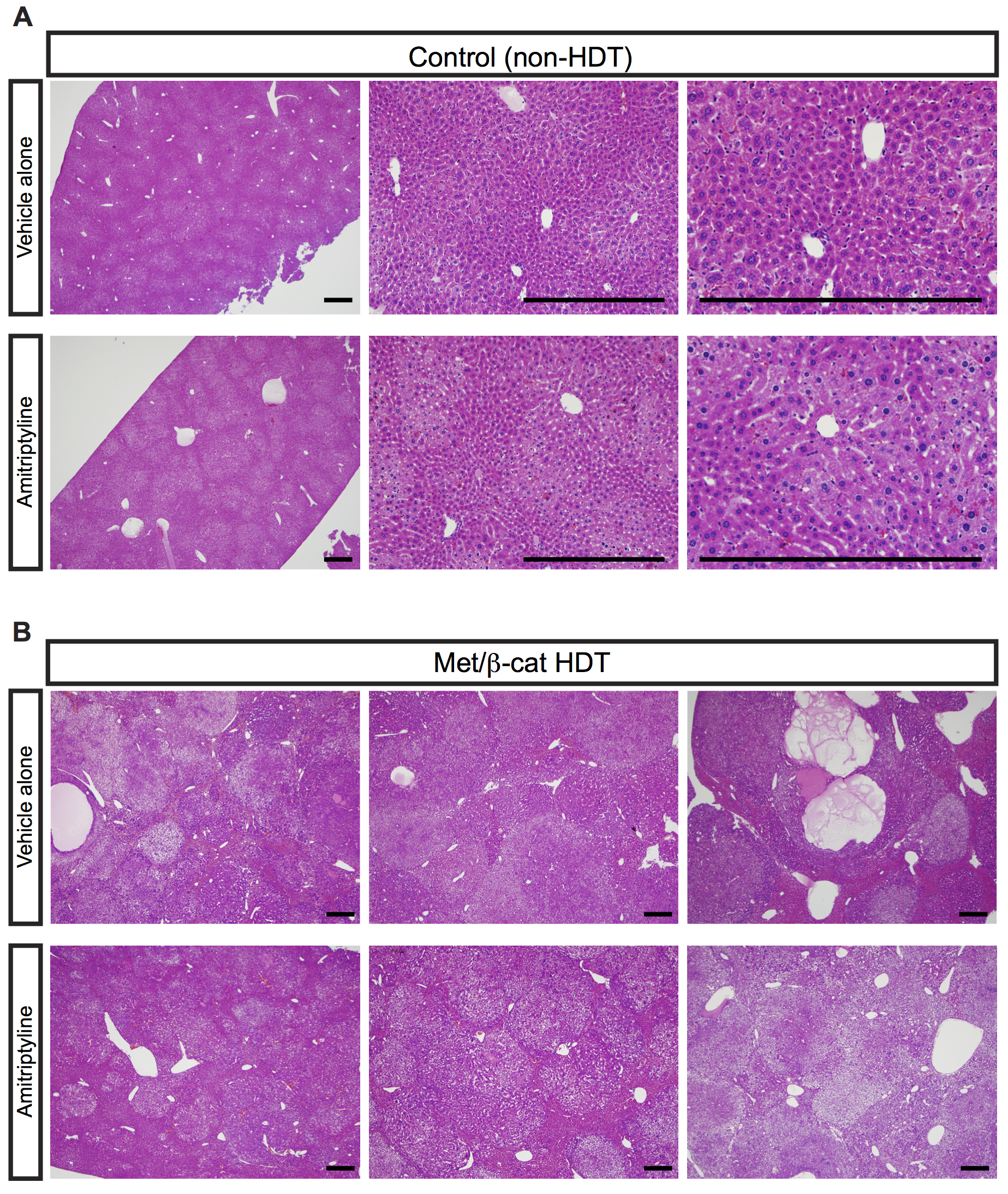

Supplement: S9 Fig — (A) Representative images of livers from control, non-hydrodynamically transfected (non-HDT) mice. Mice were treated with saccharine alone (vehicle only, top row) or amitriptyline plus saccharine (bottom row). Sections show an orderly arrangement of hepatocytes, without cytological atypia. (B) Representative images of mice hydrodynamically transfected with activated β-catenin and Met (Met/β-cat HDT). Mice were treated with saccharine alone (vehicle only, top row) or amitriptyline plus saccharine (bottom row). Sections show innumerable coalescing tumors characterized by disorganized plate architecture. Scale bars, 200 μm. (TIFF) [file pgen.1005305.s009.tiff]

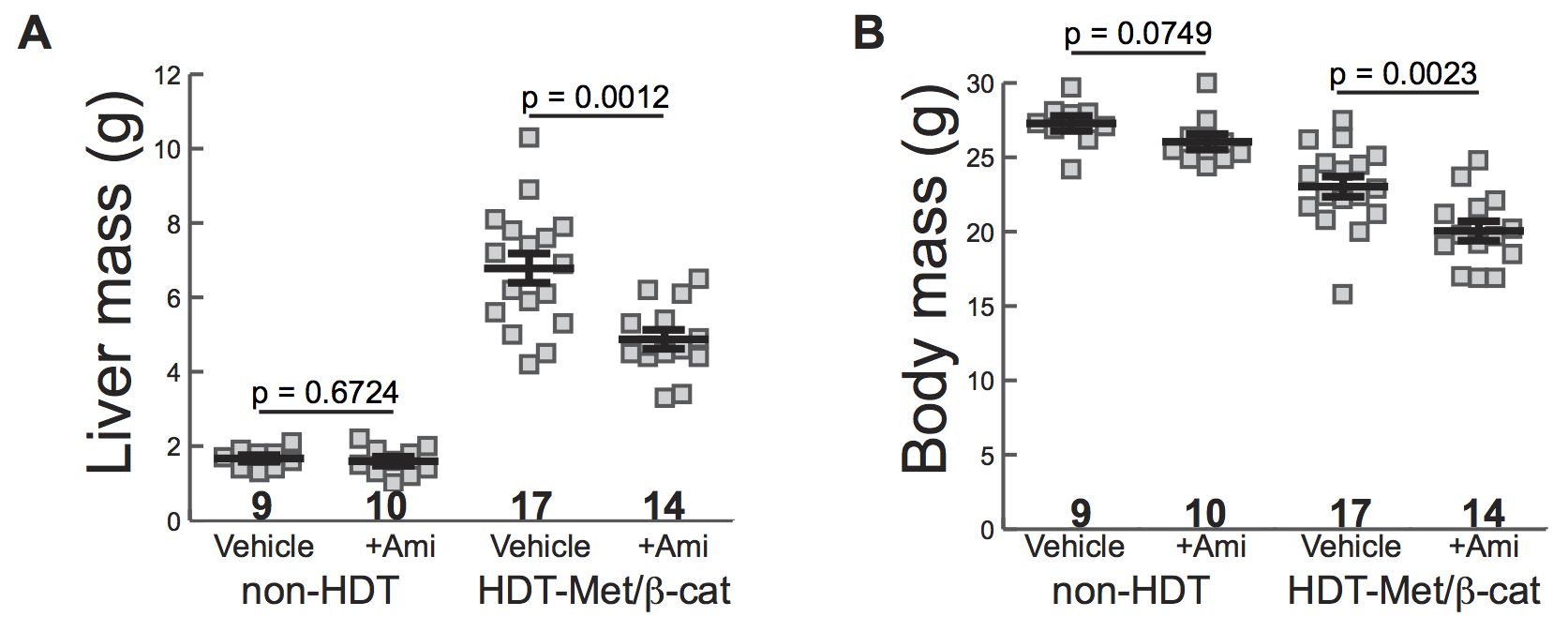

Supplement: S10 Fig — (A) Graph showing mean liver mass ± SEM for non-HDT and Met/β-cat HDT mice treated with saccharine alone (vehicle) or amitriptyline plus saccharine (+Ami). P values calculated with Mann-Whitney test. (B) Graph showing mean body mass ± SEM for non-HDT and Met/β-cat HDT treated with saccharine alone (vehicle) or amitriptyline plus saccharine (+Ami). P values calculated with Mann-Whitney test. The number of mice in each group is shown above the x-axis. (TIFF) [file pgen.1005305.s010.tiff]

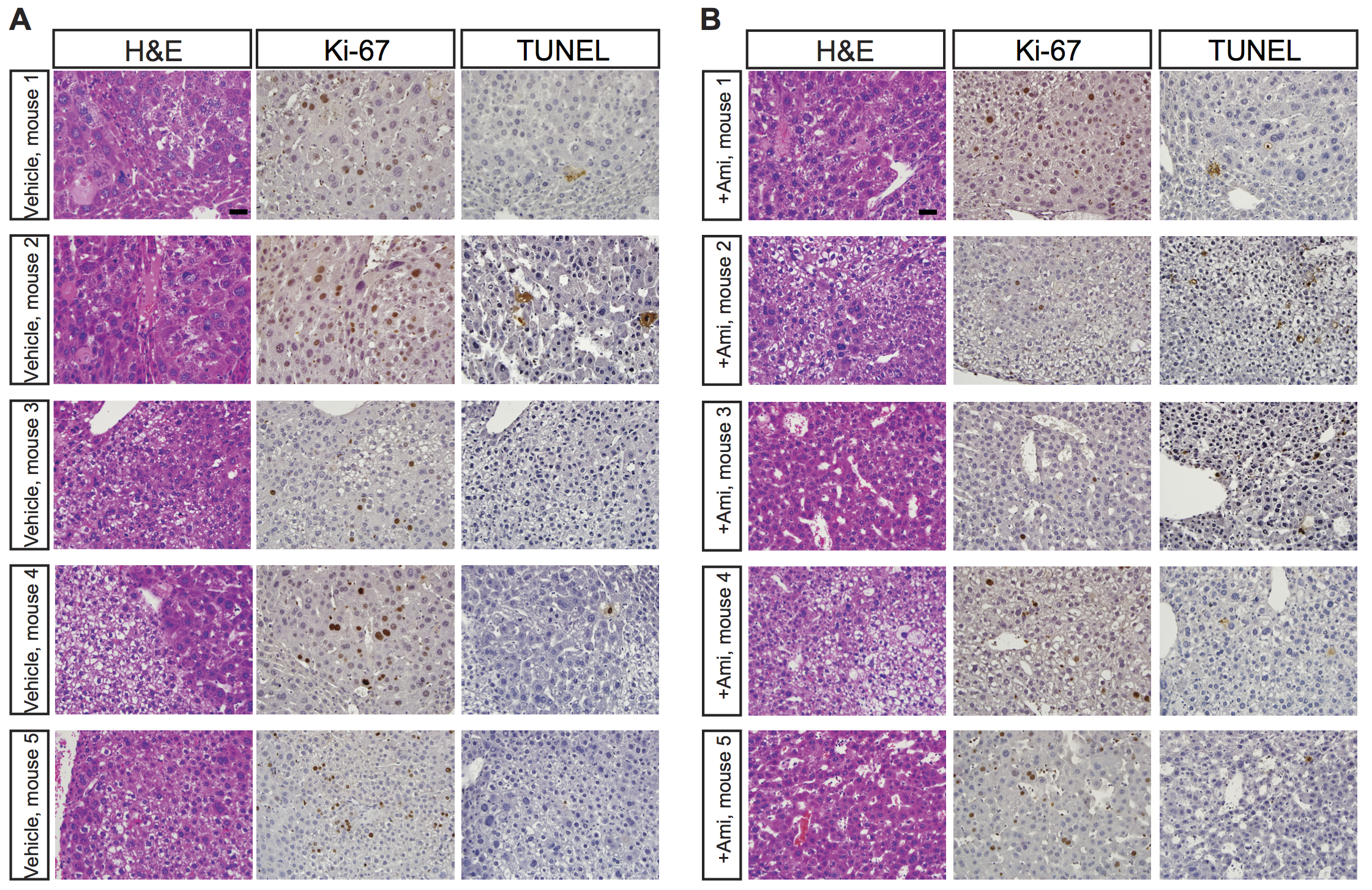

Supplement: S11 Fig — Mice were treated with saccharine alone (A) or amitriptyline plus saccharine (B). Ki-67 and TUNEL staining were performed using 3, 3'-diaminobenzidine (DAB) substrate, so positive-staining cells are brown, and hematoxylin counterstain, to highlight nuclei and other basophilic structures in blue. Scale bars, 20 μm. Quantification of this experiment is shown in Fig 6G and6H. (TIFF) [file pgen.1005305.s011.tiff]
