# Supplementary material for: Identification of Chemical Inhibitors of β-Catenin-Driven Liver Tumorigenesis in Zebrafish
Source: PLoS Genet. 2015 Jul 2;11(7):e1005305. doi: 10.1371/journal.pgen.1005305 (PMC4489858; doi:10.1371/journal.pgen.1005305)
Supplement: S1 Table — (DOCX) [file pgen.1005305.s013.docx]

**Table S1:** Transgenic zebrafish strains used in this study

| **Transgenic Construct** | **Geno-type** | **Regulatory region(s) and description** | **Coding sequence** | **Purpose used here** | **Ref** |
| --- | --- | --- | --- | --- | --- |
| *Tg(7xTCF-Xla.Siam:GFP)* | *ia4* | 7 consensus Tcf/Lef binding sites; drives expression in cells with active Wnt/β-catenin signaling | GFP; green fluorescent protein | Visualize and quantify Wnt/β-catenin activity | [1] |
| *Tg(-2.8fabp10a:*  *EGFP)* | *as3* | *fatty acid binding protein 10a, liver basic*; drives expression in hepatocytes from 72 hpf to adulthood | EGFP; enhanced green fluorescent protein | Visualize/ identify hepatocytes | [2] |
| *Tg(fabp10a:*  *rasGFP)* | *s942* | *fatty acid binding protein 10a*, *liver basic*; drives expression in hepatocytes from 72 hpf to adulthood | rasGFP; membrane-localized green fluorescent protein | Visualize/ identify hepatocyte cell membranes | [2,3] |
| *Tg(fabp10a:*  *pt-β-cat, cryaa:Venus)* | *s985, s986, s987* | 1. *fatty acid binding protein 10a, liver basic*: drives expression in hepatocytes from 72 hpf to adulthood  2. *crystallin, alpha A*: drives expression in lens of eye from 72 hpf to adulthood | 1. *Xenopus* β-catenin with 4 point mutations;  2. Venus fluorescent protein | Hepatocyte-specific expression of activated β-catenin; transgenic zebrafish identified by Venus eyes | 1. [2,4]  2. [5] |

**References**

1. Moro E, Ozhan-Kizil G, Mongera A, Beis D, Wierzbicki C, Young RM, et al. In vivo Wnt signaling tracing through a transgenic biosensor fish reveals novel activity domains. Dev Biol. 2012;366: 327–340. doi:10.1016/j.ydbio.2012.03.023

2. Her GM, Yeh Y-H, Wu J-L. 435-bp liver regulatory sequence in the liver fatty acid binding protein (L-FABP) gene is sufficient to modulate liver regional expression in transgenic zebrafish. Dev Dyn 2003;227: 347–356. doi:10.1002/dvdy.10324

3. Cheung ID, Bagnat M, Ma TP, Datta A, Evason K, Moore JC, et al. Regulation of intrahepatic biliary duct morphogenesis by Claudin 15-like b. Dev Biol. 2012;361: 68–78. doi:10.1016/j.ydbio.2011.10.004

4. Yost C, Torres M, Miller JR, Huang E, Kimelman D, Moon RT. The axis-inducing activity, stability, and subcellular distribution of beta-catenin is regulated in Xenopus embryos by glycogen synthase kinase 3. Genes Dev. 1996;10: 1443–1454.

5. Kurita R, Sagara H, Aoki Y, Link BA, Arai K, Watanabe S. Suppression of lens growth by alphaA-crystallin promoter-driven expression of diphtheria toxin results in disruption of retinal cell organization in zebrafish. Dev Biol. 2003;255: 113–127.
