# Supplementary material for: Identification of Chemical Inhibitors of β-Catenin-Driven Liver Tumorigenesis in Zebrafish
Source: PLoS Genet. 2015 Jul 2;11(7):e1005305. doi: 10.1371/journal.pgen.1005305 (PMC4489858; doi:10.1371/journal.pgen.1005305)
Supplement: S2 Table — (DOCX) [file pgen.1005305.s014.docx]

**Table S2:** Comparison of diagnostic features of human hepatocellular carcinoma (HCC) and criteria used to diagnose HCC in zebrafish

| **Human HCC feature** | **Type of criteria** | **Used for diagnosing HCC in *Tg(fabp10a:pt-β-cat)* zebrafish?** |
| --- | --- | --- |
| Architectural abnormalities: thickened cell plates, pseudoglands, or compact growth [1] | Histologic | Yes |
| Cytologic abnormalities: nuclear contour irregularities, coarse chromatin, prominent nucleoli, and/or increased nuclear-to-cytoplasmic ratios [1] | Histologic | Yes |
| Hepatoid appearance: Tumor cells resemble hepatocytes and may show cytoplasmic glycogen, fat, water, or inclusions [1] | Histologic | Yes |
| Positive staining for Arginase-1, hepatocyte paraffin-1, and glypican-3 [2] | Immunohistochemical | No (not tested) |
| Alpha-fetoprotein elevation [1] | Laboratory (serum) test | No (not tested) |

**References**

1. Misdraji J, Yerian L, Deshpande V, Kim GE, Mino-Kenudson M, Kakar S, et al. Diagnostic Pathology: Hepatobiliary and Pancreatic. First Edition. Lamps LW, editor. Manitoba, Canada: Amirsys Publishing, Inc.; 2011.

2. Timek DT, Shi J, Liu H, Lin F. Arginase-1, HepPar-1, and Glypican-3 are the most effective panel of markers in distinguishing hepatocellular carcinoma from metastatic tumor on fine-needle aspiration specimens. Am J Clin Pathol. 2012;138: 203–210. doi:10.1309/AJCPK1ZC9WNHCCMU
