# Supplementary material for: Identification of Chemical Inhibitors of β-Catenin-Driven Liver Tumorigenesis in Zebrafish
Source: PLoS Genet. 2015 Jul 2;11(7):e1005305. doi: 10.1371/journal.pgen.1005305 (PMC4489858; doi:10.1371/journal.pgen.1005305)
Supplement: S3 Table — (DOCX) [file pgen.1005305.s015.docx]

**Table S3:** Body mass, liver mass, and liver-to-body mass ratios of *Tg(fabp10a:pt-β-cat)* zebrafish and sibling controls

| **Time point**  **(Age)** | **Genotype** | **Body mass** | **Liver mass** | **Liver-to-body mass ratio** |
| --- | --- | --- | --- | --- |
| 2 months post fertilization | Non-transgenic | 0.3373 | 0.0152 | 0.045063741 |
|  |  | 0.2213 | 0.0088 | 0.039765025 |
|  |  | 0.1774 | 0.0057 | 0.032130778 |
|  |  | 0.252 | 0.0095 | 0.037698413 |
|  |  | 0.1968 | 0.0092 | 0.046747967 |
|  |  | 0.1386 | 0.0097 | 0.06998557 |
| 2 months post fertilization | *Tg(fabp10a:pt-β-cat)* | 0.1946 | 0.0121 | 0.062178828 |
|  |  | 0.2074 | 0.0163 | 0.078592093 |
|  |  | 0.1662 | 0.008 | 0.048134777 |
|  |  | 0.1972 | 0.016 | 0.081135903 |
|  |  | 0.0903 | 0.0047 | 0.052048726 |
|  |  | 0.0916 | 0.0067 | 0.073144105 |
|  |  |  |  |  |
| 3 months post fertilization | Non-transgenic | 0.349 | 0.018 | 0.051575931 |
|  |  | 0.465 | 0.0226 | 0.048602151 |
|  |  | 0.4387 | 0.0219 | 0.049920219 |
|  |  | 0.2752 | 0.0088 | 0.031976744 |
|  |  | 0.301 | 0.0147 | 0.048837209 |
|  |  | 0.2801 | 0.0093 | 0.033202428 |
|  |  | 0.1943 | 0.0081 | 0.041688111 |
|  |  | 0.2585 | 0.0159 | 0.061508704 |
| 3 months post fertilization | *Tg(fabp10a:pt-β-cat)* | 0.4367 | 0.0473 | 0.108312343 |
|  |  | 0.4342 | 0.0384 | 0.088438508 |
|  |  | 0.458 | 0.0419 | 0.091484716 |
|  |  | 0.3547 | 0.0165 | 0.046518184 |
|  |  | 0.2559 | 0.0169 | 0.066041422 |
|  |  | 0.1448 | 0.02 | 0.138121547 |
|  |  | 0.1614 | 0.0054 | 0.033457249 |
|  |  | 0.1286 | 0.0103 | 0.080093313 |

**Table S3**, continued

| **Age** | **Genotype** | **Body mass** | **Liver mass** | **Liver-to-body mass ratio** |
| --- | --- | --- | --- | --- |
| 4 months post fertilization | Non-transgenic | 0.4001 | 0.0202 | 0.050487378 |
|  |  | 0.5197 | 0.0254 | 0.048874351 |
|  |  | 0.4504 | 0.026 | 0.057726465 |
|  |  | 0.374 | 0.0369 | 0.098663102 |
|  |  | 0.5494 | 0.0278 | 0.050600655 |
|  |  | 0.5297 | 0.0392 | 0.074004153 |
|  |  | 0.4714 | 0.0313 | 0.066397964 |
|  |  | 0.3708 | 0.0276 | 0.074433657 |
|  |  | 0.3745 | 0.02 | 0.053404539 |
|  |  | 0.4295 | 0.018 | 0.041909197 |
| 4 months post fertilization | *Tg(fabp10a:pt-β-cat)* | 0.5079 | 0.0365 | 0.07186454 |
|  |  | 0.3922 | 0.0264 | 0.067312596 |
|  |  | 0.2257 | 0.0206 | 0.091271599 |
|  |  | 0.1903 | 0.0278 | 0.146085129 |
|  |  | 0.3598 | 0.0314 | 0.087270706 |
|  |  | 0.6176 | 0.0454 | 0.073510363 |
|  |  | 0.3572 | 0.0314 | 0.087905935 |
|  |  | 0.392 | 0.0274 | 0.069897959 |
|  |  | 0.122 | 0.0027 | 0.022131148 |
|  |  | 0.3983 | 0.0385 | 0.096660808 |
|  |  | 0.1182 | 0.0069 | 0.058375635 |
|  |  | 0.4211 | 0.0276 | 0.065542626 |
|  |  | 0.0604 | 0.0044 | 0.072847682 |
|  |  | 0.1545 | 0.0196 | 0.126860841 |
|  |  | 0.1568 | 0.0103 | 0.065688776 |
|  |  |  |  |  |
| 5 months post fertilization | Non-transgenic | 0.3269 | 0.0126 | 0.038543897 |
|  |  | 0.3678 | 0.0154 | 0.041870582 |
|  |  | 0.4252 | 0.0154 | 0.03621825 |
|  |  | 0.2752 | 0.0179 | 0.065043605 |
|  |  | 0.2726 | 0.0183 | 0.067131328 |
|  |  | 0.2657 | 0.0163 | 0.061347384 |
|  |  | 0.362 | 0.0102 | 0.028176796 |
|  |  | 0.453 | 0.0214 | 0.047240618 |
| 5 months post fertilization | *Tg(fabp10a:pt-β-cat)* | 0.3947 | 0.0204 | 0.051684824 |
|  |  | 0.2284 | 0.0331 | 0.144921191 |
|  |  | 0.4335 | 0.0785 | 0.181084198 |
|  |  | 0.2888 | 0.0312 | 0.108033241 |
|  |  | 0.4108 | 0.0286 | 0.069620253 |
|  |  | 0.414 | 0.0428 | 0.103381643 |
|  |  | 0.3817 | 0.0277 | 0.072570081 |
|  |  | 0.2424 | 0.0161 | 0.066419142 |

**Table S3**, continued

| **Age** | **Genotype** | **Body mass** | **Liver mass** | **Liver-to-body mass ratio** |
| --- | --- | --- | --- | --- |
| 6 months post fertilization | Non-transgenic | 0.3821 | 0.0253 | 0.066213033 |
|  |  | 0.511 | 0.0287 | 0.056164384 |
|  |  | 0.3564 | 0.0125 | 0.035072952 |
|  |  | 0.3605 | 0.0154 | 0.042718447 |
|  |  | 0.3741 | 0.0138 | 0.036888532 |
|  |  | 0.2666 | 0.0139 | 0.052138035 |
|  |  | 0.3899 | 0.0189 | 0.048473968 |
|  |  | 0.3393 | 0.0201 | 0.059239611 |
|  |  | 0.3058 | 0.0197 | 0.06442119 |
| 6 months post fertilization | *Tg(fabp10a:pt-β-cat)* | 0.4406 | 0.0471 | 0.106899682 |
|  |  | 0.267 | 0.0169 | 0.06329588 |
|  |  | 0.1263 | 0.0167 | 0.132224861 |
|  |  | 0.1611 | 0.0249 | 0.154562384 |
|  |  | 0.1571 | 0.02 | 0.127307447 |
|  |  | 0.3052 | 0.0463 | 0.151703801 |
|  |  | 0.1435 | 0.0131 | 0.091289199 |
|  |  | 0.5043 | 0.0665 | 0.131865953 |
|  |  | 0.1429 | 0.0184 | 0.128761372 |
|  |  |  |  |  |
| 12 months post fertilization | Non-transgenic | 0.5151 | 0.0303 | 0.058823529 |
|  |  | 0.4861 | 0.0264 | 0.054309813 |
|  |  | 0.4022 | 0.02626 | 0.0652909 |
|  |  | 0.4063 | 0.02596 | 0.063893675 |
|  |  | 0.5015 | 0.0219 | 0.043668993 |
|  |  | 0.5377 | 0.0294 | 0.054677329 |
|  |  | 0.4097 | 0.0255 | 0.062240664 |
|  |  | 0.3052 | 0.0099 | 0.032437746 |
|  |  | 0.3625 | 0.02226 | 0.061406897 |
|  |  | 0.359 | 0.013 | 0.036211699 |
| 12 months post fertilization | *Tg(fabp10a:pt-β-cat)* | 0.3874 | 0.06636 | 0.171295818 |
|  |  | 0.4436 | 0.04926 | 0.111045987 |
|  |  | 0.3379 | 0.06356 | 0.188102989 |
|  |  | 0.4149 | 0.0406 | 0.097854905 |
|  |  | 0.5364 | 0.0931 | 0.173564504 |
|  |  | 0.3535 | 0.04866 | 0.137652051 |
|  |  | 0.346 | 0.05766 | 0.166647399 |
|  |  | 0.4573 | 0.0391 | 0.085501859 |
|  |  | 0.3406 | 0.0378 | 0.110980622 |
|  |  | 0.3577 | 0.0554 | 0.15487839 |
