# Supplementary material for: Identification of Chemical Inhibitors of β-Catenin-Driven Liver Tumorigenesis in Zebrafish
Source: PLoS Genet. 2015 Jul 2;11(7):e1005305. doi: 10.1371/journal.pgen.1005305 (PMC4489858; doi:10.1371/journal.pgen.1005305)
Supplement: S4 Table — (DOCX) [file pgen.1005305.s016.docx]

**Table S4:** Key pathways in *Tg(fabp10a:pt-β-cat)* zebrafish and human HCC

| **Category** | **Pathway** | **Fold enrichment** | **p-value** |
| --- | --- | --- | --- |
| DNA replication | GO:0044786\|cell cycle DNA replication | 8.17545376 | 8.61E-11 |
|  | hsa03030\|DNA replication | 10.48720379 | 8.85E-14 |
|  | DNA Replication (reactome) | 3.902346347 | 7.92E-08 |
| Telomeres | GO:0032201\|telomere maintenance via semi-conservative replication | 12.56046987 | 1.57E-12 |
|  | Extension of Telomeres (reactome) | 10.80240421 | 8.81E-13 |
| DNA repair | GO:0006297\|nucleotide-excision repair, DNA gap filling | 11.95660112 | 8.88E-09 |
|  | hsa03430\|Mismatch repair | 9.831753555 | 1.11E-08 |
|  | Gap-filling DNA repair synthesis and ligation in GG-NER (reactome) | 10.91263282 | 1.14E-08 |
| Cell proliferation/ cell cycle | GO:0008283\|cell proliferation | 1.500436219 | 0.003250971 |
|  | hsa04110\|Cell cycle | 2.162985782 | 0.011864192 |
|  | Cell Cycle, Mitotic (reactome) | 1.799002875 | 0.000157059 |
| Cell motility and adhesion | GO:0048870\|cell motility | 1.776453065 | 1.04E-05 |
|  | hsa04514\|Cell adhesion molecules (CAMs) | 2.457938389 | 0.014841083 |
|  | Adherens junctions interactions | 4.243801653 | 0.012181715 |
| Cancer | hsa05200\|Pathways in cancer | 1.60221169 | 0.017459666 |
