# Supplementary material for: Identification of Chemical Inhibitors of β-Catenin-Driven Liver Tumorigenesis in Zebrafish
Source: PLoS Genet. 2015 Jul 2;11(7):e1005305. doi: 10.1371/journal.pgen.1005305 (PMC4489858; doi:10.1371/journal.pgen.1005305)
Supplement: S5 Table — (DOCX) [file pgen.1005305.s017.docx]

**Table S5:** Hit compounds that suppressed larval liver enlargement caused by activated β-catenin in zebrafish larvae.

| **Catalog Number** | **Compound Name** | **Description** |
| --- | --- | --- |
| EMD  328009 | 1-nitro-2-[(Z)-[5-(3-nitrophenyl)furan-2-yl]-  methylideneamino]guanidine | ERK Inhibitor III |
| EMD  343021 | bis(5-hydroxy-1H-indol-2-yl)methanone | Flt-3 Inhibitor II |
| EMD  361541 | 2-[(3-iodophenyl)methylsulfanyl]-  5-pyridin-4-yl-1,3,4-oxadiazole | GSK-3β Inhibitor II |
| EMD  420119 | SP600125  (1,9-Pyrazoloanthrone) | JNK Inhibitor II |
| EMD  420123 | EMD 420123  (N1-Methyl-1,9-pyrazoloanthrone) | JNK Inhibitor* |

| Sigma  P9623 | Paroxetine  ((3*S*,4*R*)-3-[(2*H*-1,3-benzodioxol-5-yloxy)methyl]-4-(4-fluorophenyl)piperidine) | Selective serotonin reuptake inhibitor (SSRI); antidepressant |
| --- | --- | --- |
| Sigma  A8404 | Amitriptyline  (3-(10,11-dihydro-5*H*-dibenzo[*a*,*d*]cycloheptene-5-ylidene)-*N*,*N*-dimethylpropan-1-amine) | Tricyclic  antidepressant |
| Sigma  F6886 | Forskolin | Activates adenylate cyclase; antihypertensive |

| ENZO  AC-1366 | Mirtazapine | Tetracyclic antidepressant |
| --- | --- | --- |
| ENZO  AC-572 | Clomipramine | Tricyclic antidepressant |

*EMD 420123 is described as “JNK inhibitor negative control” in the EMD Kinase Inhibitor I Library. The reported JNK IC50 of EMD421023 is 18-24 μM; the IC50 of SP600125 is 0.11-0.19 μM [33].
